# Cryptic diversity and speciation in an endemic copepod crustacean *Harpacticella inopinata* within Lake Baikal

**DOI:** 10.1002/ece3.11471

**Published:** 2024-05-31

**Authors:** Elena Kochanova, Tatyana Mayor, Risto Väinölä

**Affiliations:** ^1^ Finnish Museum of Natural History University of Helsinki Helsinki Finland; ^2^ Laboratory of Ichthyology Limnological Institute SB RAS Irkutsk Russia

**Keywords:** ancient lakes, Copepoda, endemism, phylogeography, speciation

## Abstract

Ancient lakes are hotspots of species diversity, posing challenges and opportunities for exploration of the dynamics of endemic diversification. Lake Baikal in Siberia, the oldest lake in the world, hosts a particularly rich crustacean fauna, including the largest known species flock of harpacticoid copepods with some 70 species. Here, we focused on exploring the diversity and evolution within a single nominal species, *Harpacticella inopinata* Sars, 1908, using molecular markers (mitochondrial COI, nuclear ITS1 and 28S rRNA) and a set of qualitative and quantitative morphological traits. Five major mitochondrial lineages were recognized, with model‐corrected COI distances of 0.20–0.37. A concordant pattern was seen in the nuclear data set, and qualitative morphological traits also distinguish a part of the lineages. All this suggests the presence of several hitherto unrecognized cryptic taxa within the baikalian *H. inopinata*, with long independent histories. The abundances, distributions and inferred demographic histories were different among taxa. Two taxa, *H. inopinata* CE and *H. inopinata* CW, were widespread on the eastern and western coasts, respectively, and were largely allopatric. Patterns in mitochondrial variation, that is, shallow star‐like haplotype networks, suggest these taxa have spread through the lake relatively recently. Three other taxa, *H. inopinata* RE, RW and RW2, instead were rare and had more localized distributions on either coast, but showed deeper intraspecies genealogies, suggesting older regional presence. The rare taxa were often found in sympatry with the others and occasionally introgressed by mtDNA from the common ones. The mitochondrial divergence between and within the *H. inopinata* lineages is still unexpectedly deep, suggesting an unusually high molecular rate. The recognition of true systematic diversity in the evaluation and management of ecosystems is important in hotspots, as it is everywhere else, while the translation of the diversity into a formal taxonomy remains a challenge.

## Graphical abstract

In this paper, we delved into the diversification and evolutionary history of endemic copepod crustacean *Harpacticella inopinata* from the ancient Lake Baikal. We found a complex of five cryptic species with deep levels of mitochondrial divergence and long independent evolutionary histories.

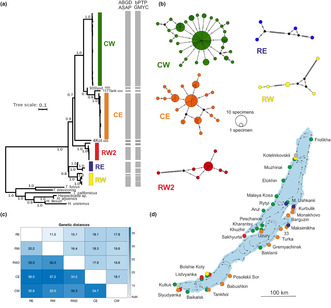

## 1 INTRODUCTION

Ancient lakes, or long‐lived lakes, are biodiversity hotspots with unique endemic faunas that evolved in isolation through long periods of time, and provide excellent settings for tracing evolutionary processes and mechanisms underlying species diversification (Kroll et al., 2012; Martens, 1997; Miura et al., 2019). While in other freshwater systems, new species are generally thought to arise allopatrically by gradual accumulation of reproductive isolation among geographically isolated populations, the suggested driving forces of speciation in ancient lakes rather include ecological specialization (Kovalenkova et al., 2020), bottleneck/founder events after climatic and geological changes (Genner et al., 2010), hybridization and introgression that promote post‐colonization adaptations and accelerate diversification (Marques et al., 2019), as well as intralacustrine geographical barriers (Mourguiart, 2000).

Lake Baikal in East Siberia is the deepest (1642 m), the most voluminous (23,000 km^3^) and the oldest (25–30 Ma) freshwater lake on Earth (Martens, 1997). The ancient origin of the lake, climatic shifts from subtropical to continental (Schluter, 2000), Pleistocene glaciations, and the evolution of oxygenated abyssal habitats promoted speciation in Baikal (Khursevich et al., 2001; Timoshkin, 2001). Among all ancient lakes, the diversity of crustaceans is the highest in Lake Baikal, both in terms of the numbers of species and their proportion of the fauna (Martens & Schön, 1999). Molecular studies of Baikalian amphipods and ostracods have exposed an additional layer of diversity in these crustaceans, frequently identifying cryptic species and deep intraspecies divergences (Daneliya & Väinölä, 2014; Gurkov et al., 2019; Moskalenko et al., 2020; Schön et al., 2017; Väinölä & Kamaltynov, 1999). A recurring concern in these studies is the timing of the evolutionary radiations. By comparing molecular distances with plausible geological and climatic events that could trigger diversification, and considering the minor morphological differences in view of prominent molecular differentiation, it has been repeatedly suggested that molecular rates in Baikal would be considerably higher than elsewhere (Daneliya et al., 2011; Gurkov et al., 2019; Naumenko et al., 2017).

Many of the Baikalian crustacean groups and radiations, however, still remain poorly studied. A prominently diverse group are harpacticoid copepods, microscopic benthic crustaceans that in general rank among the most diverse taxa in the lake, with high endemism: 67 of the 78 species recognized from Baikal are endemic (Okuneva, 1989; Timoshkin, 2001). With their benthic lifestyle, the Harpacticoida are thought to be more likely a subject of extensive radiations triggered by past changes in water levels and depths than the other, predominantly planktonic copepod orders. (Martens & Schön, 1999). Almost no molecular record of their diversification so far exists, however. The recognized harpacticoid diversity is divided between two families, of which Canthocamptidae comprises 77 species, in multiple described species flocks (Okuneva, 1989; Timoshkin, 2001).

**FIGURE 1:**
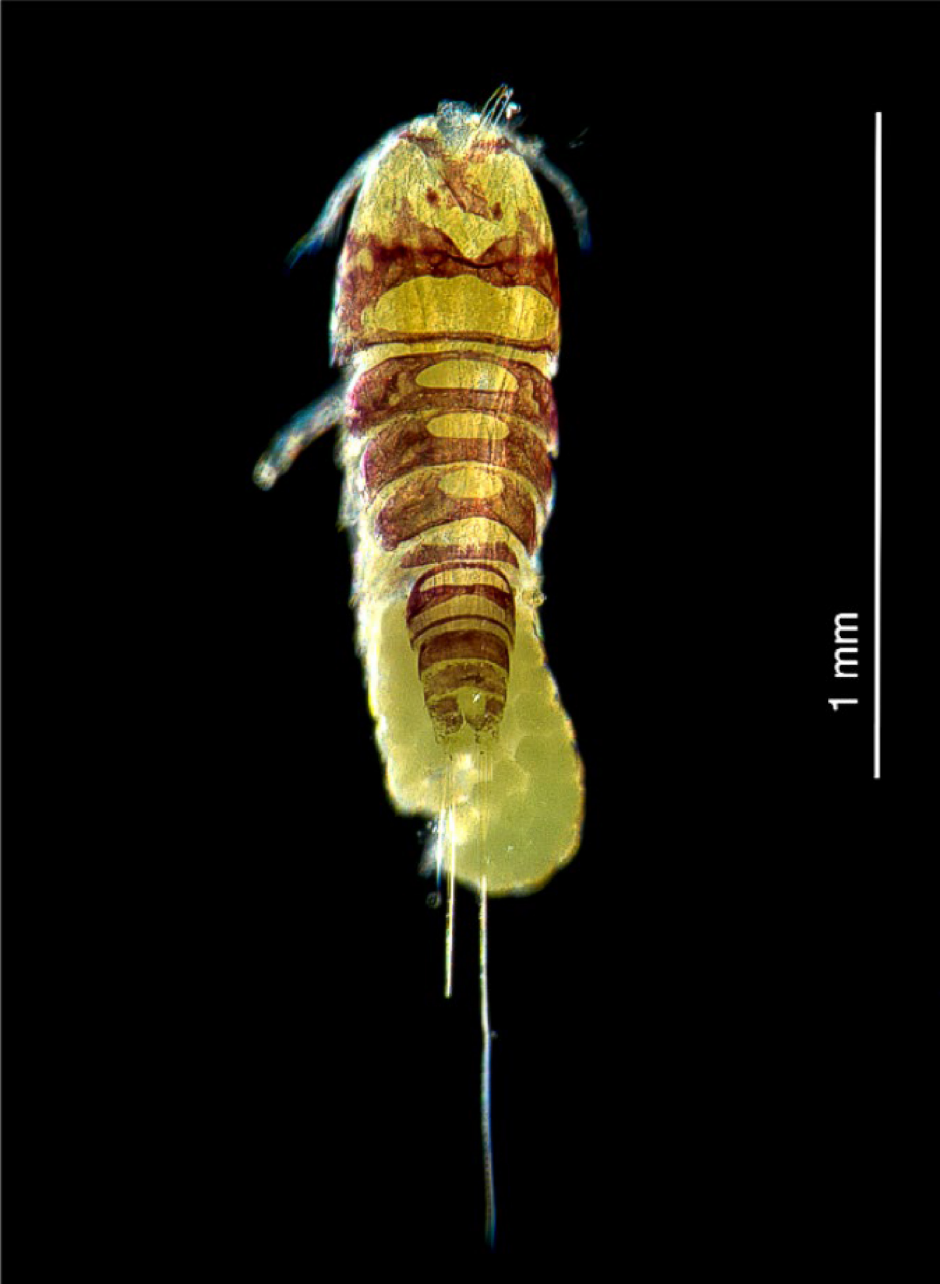
A female *Harpacticella inopinata* from the Barguzin sampling site.

The first harpacticoid described from Lake Baikal, *Harpacticella inopinata* Sars, 1908 (Figure 1), however uniquely belongs to the predominantly marine family Harpacticidae (Borutzky, 1952). *H. inopinata* is the most abundant species in the littoral zone of the lake, with densities of up to 800,000 m^−2^ (Okuneva, 1989). It is an important food source for benthic fishes, particularly for juvenile sculpins (Evstigneeva, 1986). The species is widely distributed and ubiquitous across all coastal areas of the lake (Timoshkin, 2001) and shows considerable ecological plasticity, occupying various niches and substrates such as stones, silt and sand (Okuneva, 1989). Recent studies have documented both morphological and molecular variations within the species (Evstigneeva & Sobakina, 2008; Fefilova et al., 2023), but no formal taxonomic diagnoses or integrative studies relating molecules to morphology have been presented.

In this study, we explore the true diversity of the unique Baikalian harpacticoid copepod, *Harpacticella inopinata*, in an integrative approach, and set it in the framework of the expanding comparative information of the diversity and evolution of the Baikalian fauna. We compare populations and specimens from most of the lake‐wide species range using three sequenced molecular markers (COI, ITS1 and 28S rRNA), qualitative morphological traits and morphometric measurements. The samples come from all three sub‐basins of Lake Baikal, from both Eastern and Western shores, and from different types of sediments. Particular questions include the search for cryptic taxa, their characterization by different sets of characters, phylogeographical variation, traces of demographic history, age and rate of diversification.

## 2 MATERIALS AND METHODS

### 2.1 Sampling

Harpacticoids were collected in two sampling campaigns in 2021. Ten locations in the Eastern and Northern parts of the lake were sampled in June from RV ‘Papanin’ by scuba divers at 1 to 30 m depths. The animals were sorted alive under a light microscope and fixed in 96% ethanol for subsequent analyses. Samples from 19 locations in the Western and Southern parts were collected in July from the littoral zone at 0–5 m depths using a 100 μm mesh scoop‐net. Samples were filtered multiple times using the net, fixed in 95% ethanol, sorted under the microscope and the harpacticoids were stored in 99% ethanol.

Overall, the sampling comprised 29 locations covering the shorelines of all three sub‐basins of Lake Baikal (south, central and north) as well as the Olkhon Island and Malyi Ushkanii Island (Table 1). The sampling comprised various habitats (sediment type: stones and sands) (Table 1). In each sample from each location, *H. inopinata* was present.

**TABLE 1:** Sampling locations, depths and substrates, numbers of specimens analysed morphologically and for each gene.

Locality	Depth (m)	Substrate	*N* morphology	Number of sequences		
				COI	ITS1	28S
Kharantsy, Olkhon island	0–1.5	Sand	4	4	2	1
Peschanoe, Olkhon island	0–1.5	Sand	3	3	2	–
Uzury, Olkhon island	0–1.5	Stones	4	4	1	–
Khuzhir, Olkhon island	0–1.5	Stones	4	3	2	–
Bolshie Koty	0–5	Sand, stones	16	22	7	4
Kultuk	0–1.5	Sand	5	5	1	–
Slyudyanka	0–1.5	Stones	4	7	3	1
Baikalsk	0–1.5	Stones	5	5	3	–
Tankhoi	0–1.5	Stones	3	6	6	–
Gremyachinsk	0–1.5	Sand	–	1	1	–
Babushkin	0–1.5	Stones	4	5	2	–
Turka	0–1.5	Stones	6	6	3	1
Maksimikha	0–1.5	Stones	5	6	5	2
Barguzin	0–1.5	Stones	5	7	3	–
Kurbulik	0–1.5	Sand	4	5	2	–
Monakhovo	0–1.5	Stones	4	5	3	1
Sample 33	0–1.5	Stones	4	3	2	–
Posolskii sor	0–1.5	Sand	4	3	2	–
Listvyanka	0–1.5	Stones	5	6	4	–
Elokhin	30	Sand	7	10	2	–
Tompuda	10	Sand	–	–	3	–
Malyi Ushkanii island	2–9	Sand	3	8	2	–
Sakhyurta	1	Sand	3	4	2	–
Kotelnikovskii Mys	10–22	Stones	1	3	3	–
Rytyi	10	Sand, sponge	2	5	2	–
Arul Mys	6	Sand, stones	1	2	2	–
Frolikha	30	Sponge	1	1	1	–
Malaya Kosa	7	Sand	–	3	2	–
Muzhinai Mys	10	Sand	2	2	2	–
Baklanii Mys	8	Stones	–	2	1	
Total number of specimens			109	148	74	10

### 2.2 DNA extraction, PCR amplification and sequencing

Genomic DNA was extracted using the Chelex protocol outlined in Walsh et al. (1991) and detailed in Kochanova et al. (2021). After DNA extraction, harpacticoid exoskeletons were recovered from the DNA solution and stored in 95% ethanol for further morphological examination. The primers and thermocycler protocols for three marker genes (mitochondrial COI, nuclear ITS1 and 28S) are described in Table S1 in Data S1. In addition to available copepod primers, specific *H. inopinata* primers were designed to amplify a fragment of ca 600 bp of COI gene: Hin_13F (CCT GAG TGT TCT DAT YCG GC) and Hin_630R (GTG GAG TTC AGG TTA CGG TC). The reaction mixes for each of the markers consisted of 1 μL (10 μM) of direct and reverse primers each, 12 μL of DreamTaq Hot Start PCR Master Mix (ThermoFisher Scientific), 9 μL of water without nucleases (Ambition, USA) and 2 μL of isolated DNA. PCR products were visualized by electrophoresis in 1.5% agarose gel. PCR products were then purified with ExoSap‐IT PCR Product Clean‐Up (Applied Biosystems). Sequencing was carried out using the BigDye Terminator v3.1 (Life Technology) reagent kit on the ABI PRISM 310 Genetic Analyzer (Applied Biosystems, USA).

### 2.3 Phylogenetic analysis

Bidirectional nucleotide sequences were assembled in consensus sequences in Geneious Prime 2022.2.2 (https://www.geneious.com) and checked manually. Sequences were aligned using MAFFT v7.017 (Katoh & Standley, 2016) for each gene separately and adjusted manually due to the presence of gap regions in ITS1 gene. In the nuclear ITS1 and 28S sequences, double peaks that were present in both bidirectional sequences were considered as heterozygotes and coded with an IUPAC code.

**FIGURE 2:**
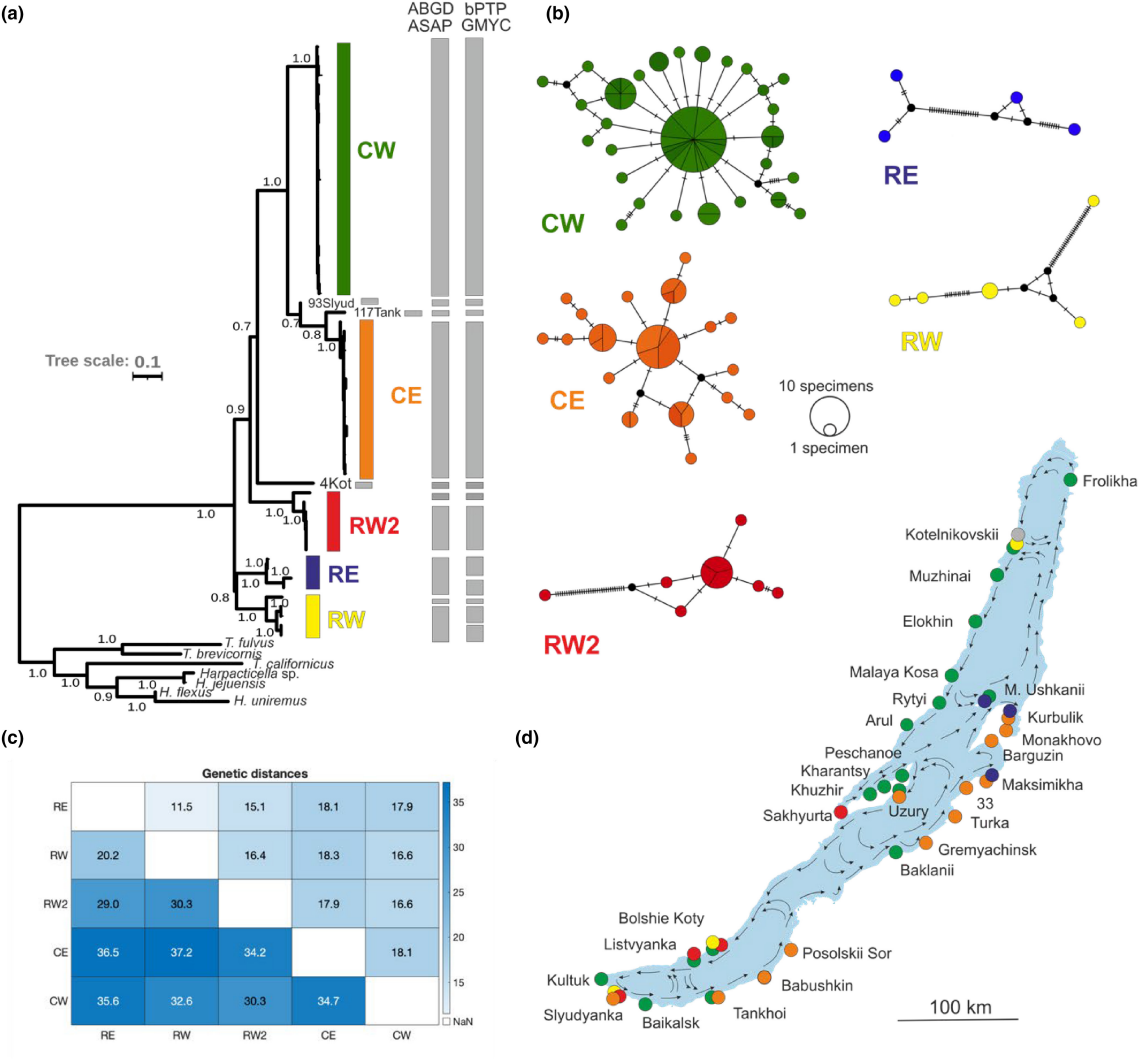
(a) A tree of relationships of unique mitochondrial COI haplotypes (ML tree with bootstrap values at the nodes), and results of various species delimitation analyses based on complete haplotype data of *Harpacticella inopinata* (each bar represents an inferred MOTU). Outgroup sequences are *Tigriopus fulvus* (MK211324), *T. brevicornis* (EF207720), *T. californicus* (AF096943), *Harpacticella jejuensis* (KM272559), *H. sp* (ZPC043‐13), *Harpacticus flexus* (MG817135) and *H. uniremus* (MH242788); (b) COI haplotype networks for each of the five main mitochondrial lineages. Crosslines denote the nucleotide differences between haplotypes. Circle size is proportional to the haplotype frequency in the data; (c) means of model‐corrected distances (below diagonal) and of p‐distances (above diagonal) (in %) between lineages from COI; (d) distribution of sampling sites (Table 1) and the observed distributions of the identified mitochondrial lineages in Lake Baikal, colour coded as in the tree. Arrows indicate surface currents.

For the mitochondrial COI, a Maximum Likelihood tree from unique haplotypes was constructed in IQ‐TREE2 software (Minh et al., 2020) using GTR + G (0.63) + I (0.32) model, which was selected as the best‐fitting model in jModelTest v. 2.1.7 (Posada, 2008). Database sequences of several other species of Harpacticidae were used as an outgroup in the analysis (see Figure 2).

The two nuclear markers are both from the multicopy rRNA gene cluster and were primarily used to test and support the systematic signal from the observed mitochondrial lineage subdivision. The 28S RNA gene is a relatively conservative and slowly evolving region that tends to provide useful information on the phylogeny among species and genera (e.g. Blanco‐Bercial et al., 2011; Khodami et al., 2017), but any simple time‐divergence relationship (clock) should not be assumed. A 28S ML tree was constructed in IQ‐TREE2 using the GTR4 + G (0.5) model, and *Tigriopus fulvus* (MN606230) as an outgroup.

The internal transcribed spacers (ITS) in turn can evolve fast and be variable at the population level both with regard to simple SNPs and to length variations (indels) (e.g. Nagy et al., 2012; Schoch et al., 2012). Since the ITSs are present in multiple copies in the genome, potentially both within and among chromosomes, they do not evolve as independent loci but can undergo inter‐copy recombination, and individuals can theoretically feature several alleles, in unequal frequencies (Denduangboripant & Cronk, 2000; Feliner & Rosselló, 2007; Göker & Grimm, 2008). Apart from the evolutionary and ensuing technical complications (co‐amplification of several copies, of potentially variable length) that would challenge the analysis even of a dichotomous diversification, the scenarios in this study involve the additional level of potential inter‐lineage reticulation. Basically, our working hypothesis for interpreting ITS variation is that the nuclear genes have evolved in geographical isolation as with and along with the mitochondria, accumulating lineage‐specific characteristics, and we are testing whether the congruence has been conserved when the lineages later have met, or whether there are signals of incongruence either at the inter‐individual or intra‐individual level, which could result from hybridization and introgression among the lineages, and have bearings on the systematic or taxonomical interpretation of the variation. A presence of different length variations in an individual is expected to blur the sequencing signal downstream of the indel region, and be seen in multiple peaks and in a general decrease of the peak strength in the sequencing chromatogram. In practice, we tried to pick up sets of ITS chromatograms as ‘clean’ as possible, which would coincide with given mitochondrial lineages from regions where they are allopatric, and hypothesized they diverged together. Specifically, we recognized consistent, lineage‐specific patterns of indel distribution. In total, 74 ITS1 gene sequences were produced (Figure S2 in Data S2).

For a technical hierarchical illustration of the ITS1 variation, we used an MP (maximum parsimony) algorithm implemented in PAUP 4.0 (Swofford, 2003). Heteroplasmic positions with double peaks were IUPAC‐coded. Indels were coded as a fifth character state using the simple indel coding algorithm in FastGap (Löytynoja & Goldman, 2008), which converts all indels with different starting and/or end positions to a matrix of binary presence/absence characters. Indels falling within the range of a longer indel were coded as unknown character states. A subset of the more clean sequences that were easily aligned and had few ambiguities were included in the primary MP analysis, from 27 specimens comprising several representative sequences for each lineage. The trees were visualized in Interactive Tree Of Life (iTOL) v5 (Letunic & Bork, 2021). A tree from all 74 sequences was also constructed and is presented in Figure S3 in Data S2.

To display the relationships of individual COI haplotypes, median‐joining haplotype networks were constructed in POPART 1.7 (Leigh & Bryant, 2015) for each of the major lineages separately. The homoplasy level parameter was set at the default value ε = 0.

For a mechanistic ‘taxon delimitation’ from COI gene sequence data, we applied four commonly used approaches. Of these ABGD (Puillandre et al., 2012) and ASAP (Puillandre et al., 2021) are distance‐based analyses, which were performed on the webserver Atelier de BioInformatique, France (https://bioinfo.mnhn.fr/abi/public/abgd/abgdweb.html and https://bioinfo.mnhn.fr/abi/public/asap/). As the GTR substitution model is not available for these methods, we used simple p‐distances. GMYC is a coalescence model approach, implemented in the ‘splits’ package (Fujisawa & Barraclough, 2013); an ultrametric tree obtained in the BEAST 2.5.2 programme (Bouckaert et al., 2014) as the input (Figure S4 in Data S2). The PTP method (Poisson Tree Processes) in turn is implemented in bPTP (Kapli et al., 2017) on the webserver of Heidelberg Institute for Theoretical Studies, Germany (https://species.h‐its.org/ptp/). An ML tree was used as the input tree.

### 2.4 Diversity statistics and demographic reconstructions

Several lineages of *H. inopinata* defined by their mitochondrial DNA were observed in this study (see Section 3), and diversity was primarily described between and within those lineages (MOTUs). Standard diversity statistics for within‐lineage variation, including the number of haplotypes (*K*), number of segregating sites (*S*), haplotype diversity (*h*) and nucleotide diversity (π) were calculated in DnaSP v6.12.03 (Librado & Rozas, 2009). Signatures of demographic changes were investigated using neutral equilibrium tests such as Tajima's (1983) *D*, Fu's (1997) Fs, and Ramos‐Onsins and Rozas's (2002) R2 for each lineage separately in DnaSP. Significant negative *D* and Fs values indicate an excess of recent mutations and can be interpreted as signatures of population expansion.

We also plotted site mismatch distributions for each lineage (frequency of pairwise nucleotide‐site differences between sequences). Distributions were compared with expectations from a stable population with constant size, and from the growth‐decline models as implemented in DnaSP, and Harpending's (1994) raggedness index *r* was used to evaluate the fit to the latter model (10,000 bootstrap replicates). The timing of a population expansion under a sudden expansion model was estimated using units of mutational time (τ) and converted to years from *t* = τ/(2μk) where μ is the mutation rate per site per year (see below) and k is the sequence length. We also used the coalescent‐based Bayesian Skyline Plot (BSP) method implemented in BEAST v2.7.3 (Drummond et al., 2012) to display an estimated population size trajectory of each lineage with *N* > 10, based on the same COI data. We used GTR + G (0.6) + I (0.2) model and an uncorrelated lognormal model. The convergence of runs was confirmed with Tracer v1.6. The plots were visualized in R‐Studio (R Core Team, 2021).

To consider the actual timing of demographic and evolutionary events on the basis of observed molecular divergence, no direct fossil‐based calibrations of Baikalian harpacticoid or of copepod rates at large are available. Therefore, to facilitate discussion, we will refer to a range of general external crustacean COI rates, 1.4% to 3.86% divergence Ma^−1^ (Ketmaier et al., 2012; Marino et al., 2011; Marrone et al., 2013), represented by a mean of 2.6% Ma^−1^, and in parallel to a hypothesis of considerably higher rates, arising from comparable studies of Baikalian amphipods (Daneliya et al., 2011; Naumenko et al., 2017), and represented by the fivefold 13% Ma^−1^.

### 2.5 Morphological analysis

Exoskeletons recovered after DNA extraction were used for further morphological analysis. Specimens were dissected under a stereo microscope and each specimen was mounted on a separate slide in glycerine as a voucher. The morphological examination was done under a Leica DM 4000B microscope, measurements were made using an ocular micrometre. Draft drawings of morphological structures were made on printed photographs, and the final drawings were prepared using the CorelDraw software.

Morphological variation was analysed from two data sets. First, a set of qualitative traits of the armature of appendages was scored, including the number of segments of Antenna I (Figure S7 in Data S2), and setation patterns of exopod Antenna II (Figure 6) and pairs of swimming appendages (P1, Figure 6; P2–P4, Figure S7 in Data S2; P5, Figure 5). Second, for a quantitative morphometric analysis, six linear measurements were made: the total lengths and widths of a single P5 endopod and of a P5 exopod, and the caudal ramus (Figure 5). We also examined the three relevant length‐to‐width ratios from these measurements. Measurements of the total length and width of the abdomen were not included since ethanol preservation can potentially cause retraction of the somites. The full matrices of both qualitative and quantitative traits are in Table S2 in Data S1. The traits were chosen for the study due to the previous records of intra‐species variability in their segmentation and setation patterns (Borutzky, 1952) and in morphometry (Lajus et al., 2015) in other copepods.

Morphometric differences were explored between (1) the molecular lineages (putative taxa) identified in this study, and (2) between the main habitat types or substrates, that is, stones and sand using two‐way ANOVA (individual samples collected from mixed substrates of sand and stones and of sand and sponges (Table 1) were excluded from the data set). Pairwise post hoc Tukey tests were then used to identify the lineages involved in the differences. To further illustrate the relative importance and relationships of the two factors (lineage, substrate) in differentiation, we constructed random forest regression trees for each morphometric variable (Bielby et al., 2010), using the R packages ‘Party’ and ‘randomForest’ (Strobl et al., 2008).

Finally, principal component analysis (PCA) of the quantitative morphometric measurements was used to illustrate the main patterns of variation and to examine the interrelationships among the measurements. The analysis was performed on the correlation matrix in Past 4.03. The loadings of individual variables on the PCs were described by simple correlations between the variables and the component scores; similar correlations were calculated for the ratios of measurements.

## 3 RESULTS

### 3.1 Molecular sequence data

From the 29 sampling locations, sequences of mitochondrial COI were obtained from 148 individuals, of ITS1 from 74, and of 28S from 10 individuals. The analysed alignments of COI, ITS1 and 28S were of 500, 340 and 685 bp length respectively. Variation of the COI data set was very high, with 199 variable sites (39.8%) within the ingroup, while ITS1 and 28S contained 64 (19.4%) and 24 (4.6%) variable sites respectively. The COI and 28S gene sequences were deposited in GenBank under accession numbers OR506752–OR506893 and OR509834–OR509843 respectively. Due to the complex nature and ambiguities of interpreting ITS1 variation (see Section 2), one representative sequence per major clade was submitted to GenBank (OR826812–OR826816), while the full alignments are available in the Dryad repository (http://doi.org/10.5061/dryad.qrfj6q5nr). Full information on the samples (location data with coordinates, list of COI haplotypes, GenBank and museum voucher IDs) is presented in Table S1 in Data S1.

### 3.2 Mitochondrial lineage diversity

Five major well‐supported mitochondrial clades or lineages within *Harpacticella inopinata* were recognized in the COI data, with different but not disjunct geographical distributions. Two of these lineages were common and widespread, and three others rare and with more localized distributions. We assign them labels that reflect their commonness and main distribution. The two abundant ones are termed the CE lineage (Common Eastern) and CW lineage (Common Western) respectively. The three more rare ones with scattered ranges are termed the RE, RW and RW2 lineages (Figure 2a). The common CE and CW made a clade of sister lineages in the ML tree, RE and RW made another clade, while RW2 was sister to the major (CE, CW) clade, and these relationships were relatively well supported (bootstrap >75%). Applying any of the ‘species delimitation’ methods (ABGD, ASAP, bPTP and GMYC), each lineage would qualify as a separate ‘species’, or rather a MOTU (molecular operational taxonomic unit). The various methods produced almost identical groupings, with minor differences between ABGD/ASAP and bPTP/GMYC regarding the RE and RW lineages (Figure 2a). Model‐corrected distances between the five main lineages varied from 20% to 37% and uncorrected distances (p‐distances) from 11% to 18% (Figure 2c).

In addition, there were several unique sequences that represented separate MOTUs (Figure 2b): two intermediate sequences between the CW and CE lineages, a single sequence from Kotelnikovskii Mys, and individual sequences clustering with(in) the RW, RW2 and RE lineages. Due to the paucity of information, these variants will not be considered further in discussion of the molecular diversity.

### 3.3 Nuclear markers

The nuclear markers were principally used to support and test the interpretation of the mitochondrial data, that is, to verify the presence of the lineage subdivision in the nuclear genome and evaluate its systematic relevance.

**FIGURE 3:**
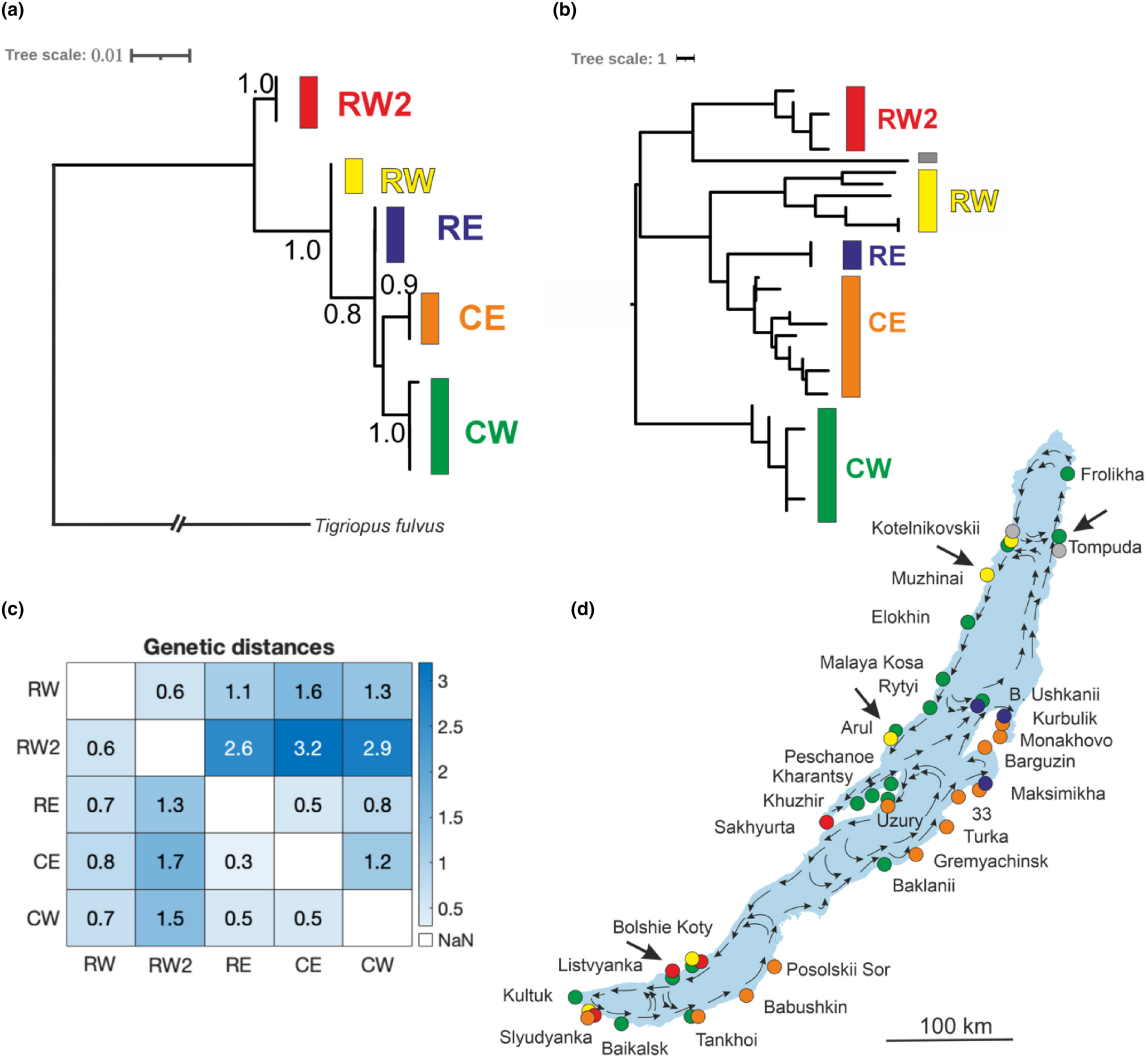
(a) ML tree from 28S rRNA sequences of *Harpacticella inopinata*, with bootstrap support values; (b) an MP tree from a pruned and gap‐coded ITS1 alignment, built‐in PAUP using ACCTRAN optimization; tree scale indicates number of differences; (c) mean model‐corrected distances (below diagonal) and mean p‐distances (above diagonal) (in %) between lineages from 28S rRNA; (d) distributions of the ITS1 lineages in Lake Baikal, strong arrows point to samples where individuals with a discrepancy in their COI versus ITS1 identities were observed; arrows within the lake indicate surface currents.

Only a few individuals were sequenced for the conservative 28S rRNA (1–3 per lineage), and their relationships were consistent with their separate lineage identities in the mtDNA marker (Figure 3a). The differences among the 28S clusters were rather small, ranging from 5 to 25 substitutions (or 0.5% to 3.2% divergence, Figure 3b). Also here, the CE and CW lineages grouped together as a clade, whereas RE and RW did not (unlike in COI), and RW2 appeared basal in the tree.

The length of ITS1 nucleotide sequences varied among individuals, and corresponded largely to their COI lineage identities: 340 bp in CW, 336 bp in CE, RE, RW2 and 325–330 bp in RW lineage. The CW sequences covered the whole length of the alignment, and eight separate gaps (indels) were present in CE, RE, RW and RW2 lineages. Most of the indels were located in the first 75 nucleotides. An MP tree with gaps coded as a fifth character state showed a separation into five groups similar to that from COI, and largely matching the individual COI lineage assignment (CE, RE, CW, RW, RW2). The topology was however different. A few mismatches in individual assignment to the ITS1 and COI clusters were seen in samples from the sites Muzhinai (1Mz, 2Mz), Arul (1Ar), Kotelnikovskii (2Kt) and Listvyanka (240, 245) which belonged to the RW and RW2 clusters of ITS1 but to CW in COI. The alignment of the full list of sequences and their affiliations to the lineages is presented in Figure S2 in Data S2.

The topology of an MP tree of concatenated data of all three markers, on representative individuals of each lineage, was similar but not identical to the COI tree in Figure 2 (Figure S11 in Data S2).

### 3.4 Diversity and distribution of individual MOTUs

The five lineages showed very dissimilar patterns of internal diversity in mtDNA. Generally, the rare lineages RW2, RW and RE were internally quite heterogeneous (nucleotide diversities π = 1.1%–4.3% haplotype diversities Hd = 0.79–1.00), whereas the widespread CE and CW lineages were much less diverse (π = 0.32%, 0.47% and Hd = 0.58, 0.76 respectively) (Table 2). The haplotype networks of CW and CE lineages showed star‐like topologies and shallow genealogies, with a common core haplotype in the centre and rarer ones separated from it by one to four mutation steps (Figure 2b). The RW2 network consisted of seven haplotypes in a star‐like topology, but one very distant haplotype separated by 32 mutation steps. The rare RE and RW lineages only comprised four and five haplotypes, almost all of them unique to a single individual, and generally distant from each other, with up to 34 mutation steps separating them.

**TABLE 2:** Molecular diversity statistics for the five main mitochondrial lineages of the *Harpacticella inopinata* complex from the 500‐bp COI gene fragment.

Lineage	*n*	*S*	π (%)	Hd	*K*	Tajima's *D*	Fu's Fs	R2	rg	“Expansion time” ma	
										2.6% (1.4%–3.9%)	13% (7%–19%)
CW	79	25	0.33	0.59	17	−2.3*	−17.1*	0.05*	0.08	0.36 (0.19–0.54)	0.07 (0.04–0.11)
CE	46	22	0.48	0.76	18	−1.7*	−10.1*	0.03*	0.02	0.82 (0.44–1.20)	0.16 (0.09–0.24)
RW2	14	35	1.12	0.89	7	−2.2*	0.8	0.22	0.09	0.31 (0.17–0.46)	0.06 (0.03–0.09)
RW	6	17	1.87	0.90	5	−0.7	1.5	0.26	0.15*	–	–
RE	4	35	4.34	1.00	4	1.1	1.2	0.24	0.44*	–	–

*Note*: Numbers of specimens (*n*), segregating sites (*S*) and haplotypes observed (*K*), estimates of haplotype diversity (Hd), nucleotide diversity (π), Tajima's *D*, Fu's Fs, Ramos‐Onsins & Rozas' R2, Harpending's raggedness index of mismatch distribution (rg) and demographic expansion times based on a range of rates from other crustaceans studies (1.4%–3.86% Ma^−1^) and a fivefold higher range (7%–19% Ma^−1^). Significant values (*p* < .05) are marked with asterisk.

The geographical distributions of the lineages showed both allopatric and sympatric patterns of occurrence (Figures 2d and 3d). Particularly, the abundant CE and CW lineages were mostly distributed along the Eastern and Western shores of the lake, respectively, with few exceptions of sympatry in Tankhoi and Uzury and the presence of western lineage/s on the Eastern shore in Baklanyi and Ushkanii islands. In the variation within these common lineages, no clear geographical structure (clustering by region or locality) was observed (Figure 2a,b; Table S1 in Data S1).

The R lineages were mainly found in sympatry with the more common C lineages, but among themselves showed an East–West phylogeographical pattern. The RE lineage was only found locally near the Svyatoi Nos peninsula in the East, while RW and RW2 were distributed along the Western shore, RW2 being restricted to the southwestern corner of the lake and in Sakhyurta, Maloe More. Within RE, specimens from Maksimikha made one cluster and those from Kurbulik and Ushkanii islands another one. Each specimen had a unique haplotype (Figure 2b). Within the RW lineage, haplotypes from Slyudyanka and Bolshie Koty formed two sister clades and that of a single Kotelnikovskii specimen was distinct from others. As with RE, almost all RW specimens had unique haplotypes. Within RW2, specimens from Listvyanka, Bolshie Koty and Sakhyurta grouped together with a star‐like haplotype configuration (Figure 2b), while the single Slyudyanka haplotype deviated from all others by 33 mutation steps (Figure 2b).

### 3.5 Projections of demographic history

**FIGURE 4:**
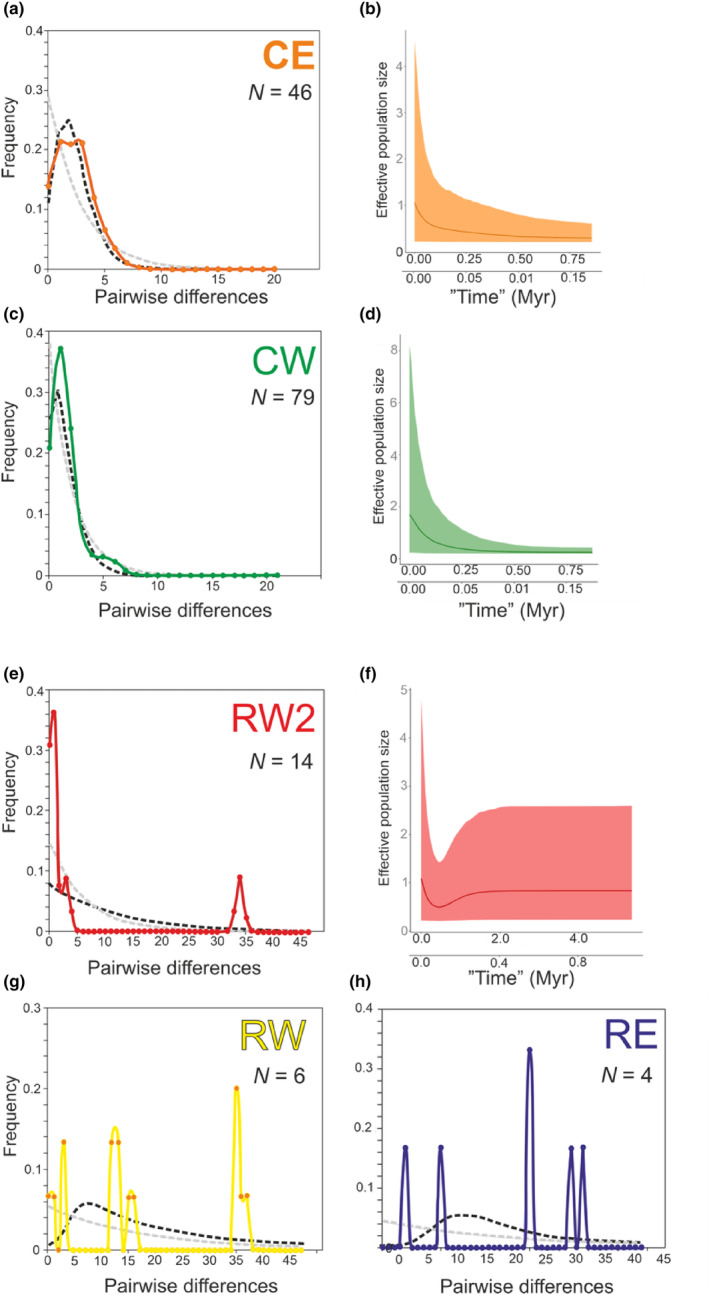
Haplotype mismatch distributions (a, c, e, g, h) and Bayesian skyline plots (b, d, f) from the five main COI lineages of *Harpacticella inopinata* (*N*, number of sequences). Observed frequencies (coloured line) were compared with the expectations from a model of stable population size (grey dashed line) and the growth‐decline model (black dashed line) obtained by DNAsp. BSPs represent changes in effective population size over time. The solid line depicts the median estimate, and the shaded area represents the 95% posterior density interval. Tentative alternative time scales correspond to COI divergence rates of 2.6% Ma^−1^, and a fivefold higher rate of 13% Ma^−1^. Note different scales on horizontal axes in a–d versus e–h.

The different genealogies of the common and rare lineages were accordingly reflected in the interpretation of their demographic histories. All three neutrality statistics (Tajima's *D*, Fu's Fs and Ramos‐Onsins & Rozas R2) indicated deviation from a mutation‐drift equilibrium in the CE and CW lineages, and an indication of population expansion in a negative *D*. The rare RW2 lineage also showed negative and significant *D*, but RE and RW did not (Table 2). Mismatch distributions were clearly unimodal in the CE and CW lineages (Figure 4a,c), but bimodal in RW2 (Figure 4e), and multimodal in RW and RE (Figure 4g,i). The observed distributions were compared with expectations under a model of a stable population with constant size (C) and under the growth‐decline (GD) model. The departure of the RE and RW lineages from the latter model of demographic expansion was evident from their Harpending's rg statistics (Table 2), whereas deviations for CE, CW and RW2 were small and insignificant, and there was a relatively good fit to the growth‐decline (expansion) model. Demographic expansion times were evaluated against the ‘conventional’ and hypothetical fivefold molecular rates (2.6% and 13% Ma^−1^, respectively, see Section 2), and would be 0.36 Ma for CW, 0.82 Ma for CE and 0.31 Ma for RW2 assuming conventional rates, or with the higher rates, 0.07, 0.16 and 0.06 Ma, respectively, (Table 2). In accord, Bayesian Skyline Plots displayed a late progressive increase in effective population size in the CE and CW lineages (Figure 4b,d), while RW2 showed a relatively constant population size through time and a recent increase (Figure 4f).

### 3.6 Morphological and morphometric diversity

Exoskeletons of 109 adult females, representing all five molecular lineages (52 from CW, 43 from CE, 8 from RW2, 3 from RW and 3 from RE) were examined in the morphological study. The numbers are lower than the number of sequences since not all exoskeletons remained undamaged after the DNA extraction. The voucher exoskeletons were deposited in the collection of the Finnish Museum of Natural History. Voucher numbers and primary morphological data are in Tables S1 and S2 in Data S1.

**FIGURE 5:**
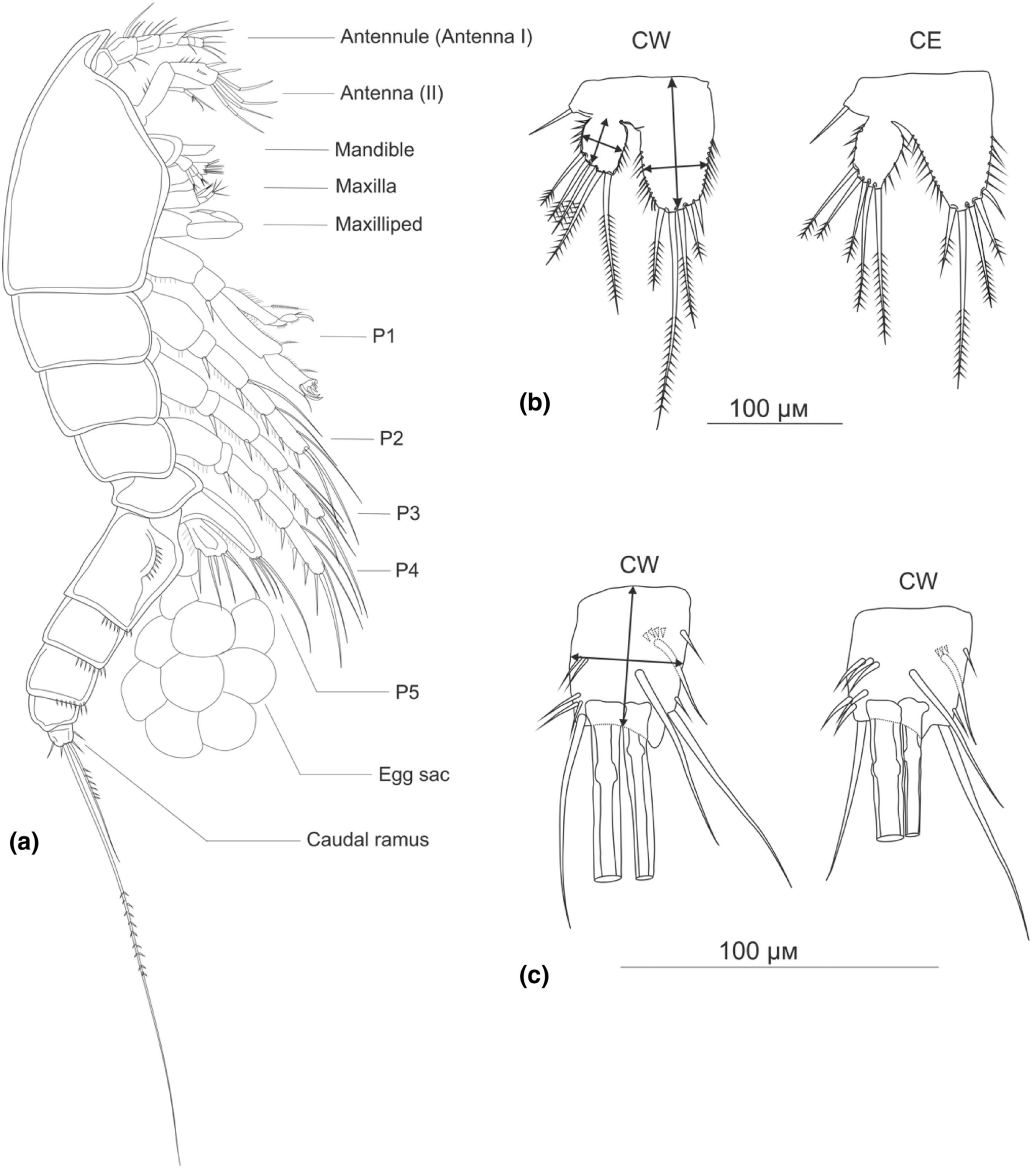
Morphology of *Harpacticella inopinata*, and definition of morphometric measurements. (a) Gross morphology: lateral view of an adult female. The length of the body is ca. 1 mm; (b) a single P5 leg from each of two CE lineage specimens from the Barguzin site (162, 168); (c) one caudal ramus from each of two CW lineage specimens from Kharantsy (5, 7). Arrows show the length and width measurements for morphometric analyses.

The external morphology of *Harpacticella* is outlined in Figure 5. Broadly, the morphology of all specimens agreed with the species descriptions of *H. inopinata* in Sars (1908), Borutzky (1952) and Okuneva (1989). However, there were also deviations from the previous descriptions, and some morphological differences were seen between the lineages in the qualitative characters, while the patterns were not completely consistent among characters.

**FIGURE 6:**
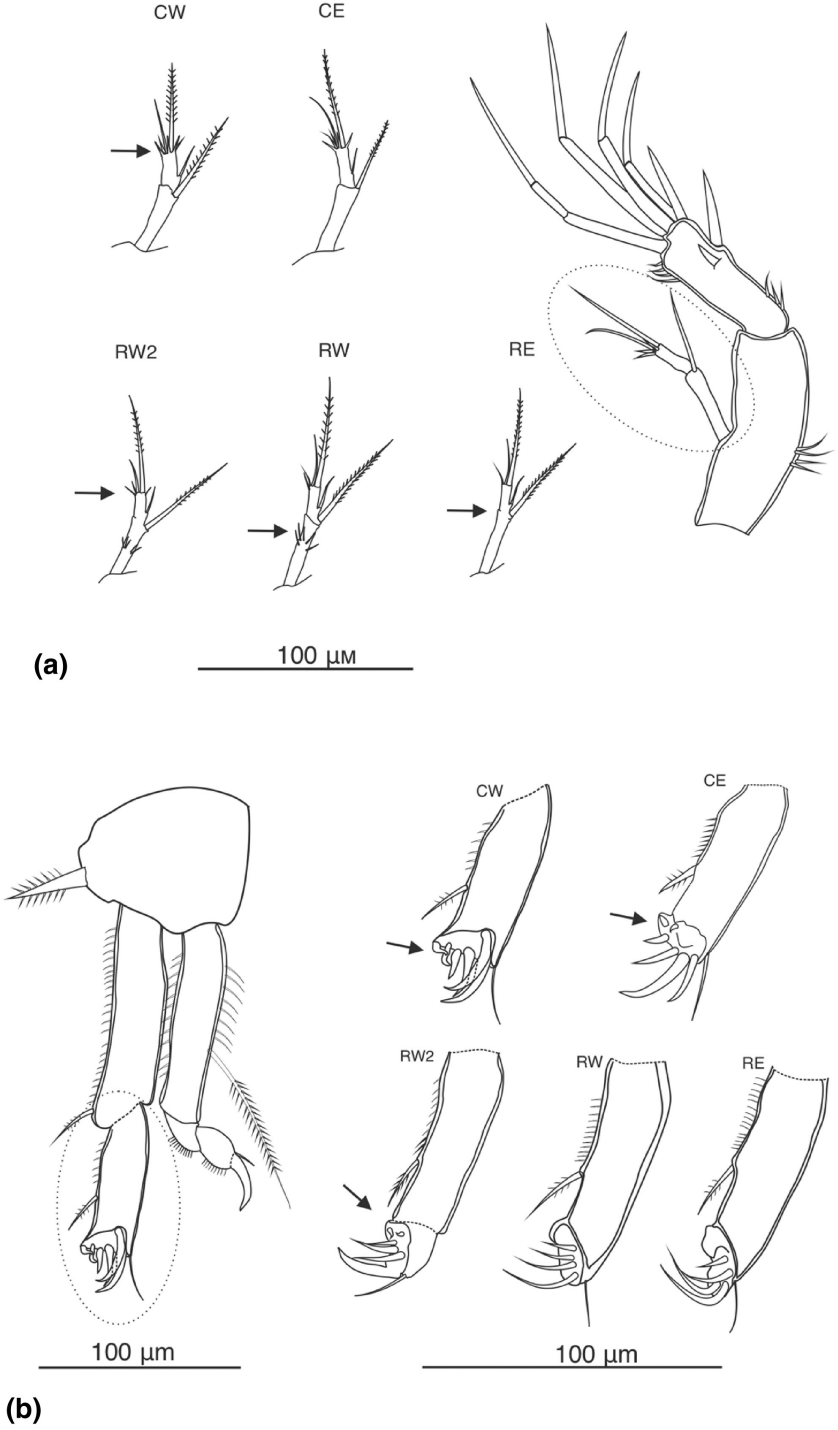
Inter‐lineage variation in qualitatively assessed morphological characters (a) Antenna II exopod; (b) P1 endopod, armature of the terminal segment. CW—specimen (7) from Kharantsy, Olkhon island, CE—Barguzin (162), RW2—Sakhyurta (9a), RW—Kotelnikovskii Mys (3kt), RE—Malyi Ushkanii island (6U). The examined segments are encircled.

The most consistent division in the qualitative characters was between the common CE + CW lineages versus the others, RW + RW2 + RE. In the Antenna II exopod, CE and CW have a ‘crown’ of 4–6 short and relatively stout spinules at the tip of the terminal segment, along with one longer seta and one feathered spine (Figure 6a). By contrast, the RE, RW and RW2 lineages only have 2–3 thin spinules at the tip together with the longer seta and feathered spine. In addition, some specimens of RW and RW2 had five thin spinules on the first segment of the AII exopod, which were not seen in the other three lineages. RW and RW2 are also distinguished from the others in that the two segments of the exopod appear fused, while they are clearly separate in the other lineages. Another division was seen in the terminal endopod segment of the first swimming appendages (P1) (Figure 6b). This segment generally bears four sharp curved claw‐like spines, but in the CE, CW and RW2 lineages there were two additional small rudimentary spines, which were absent in RW and RE.

For the morphometric data, the two‐way ANOVA to assess the differences between molecular lineages and between stone and sand substrates showed a significant effect of lineage on all measurements, that is, lengths and widths of caudal rami, P5 endopod and exopod, also on the length‐to‐width ratios of P5 endopod and exopod (*p* < .02; Table S3 in Data S1). Post hoc Tukey tests indicated that the clearest differences were generally between easterly and westerly distributed lineages (combinations of CE, RE vs. CW, RW, RW2), and the measurements of the western lineages were on average larger than of the eastern ones (Table S3 in Data S1; Figure S9 in Data S2). Similarly, the basic lineage division in the random trees analysis was often between eastern versus western lineage groups (Figure S10 in Data S2).

Substrate type also had a significant effect on the length and width of the caudal ramus and on P5 endopod length, with this effect dominating over the lineage effect in the random trees analysis (Table S3 in Data S1; Figure S10 in Data S2). These measurements were on average larger in the stones than in the sand habitat. There was a significant interaction between the effects of lineage and substrate in the length of the P5 endopod and in the length‐width ratios of P5 endopod and P5 exopod (*p* < .05; Table S3 in Data S1). No connection between habitat type and phylogenetic structure/lineage identification was found in our analysis as specimens from both sand and stone environments were mixed in the clades (Figure 2a,d).

**FIGURE 7:**
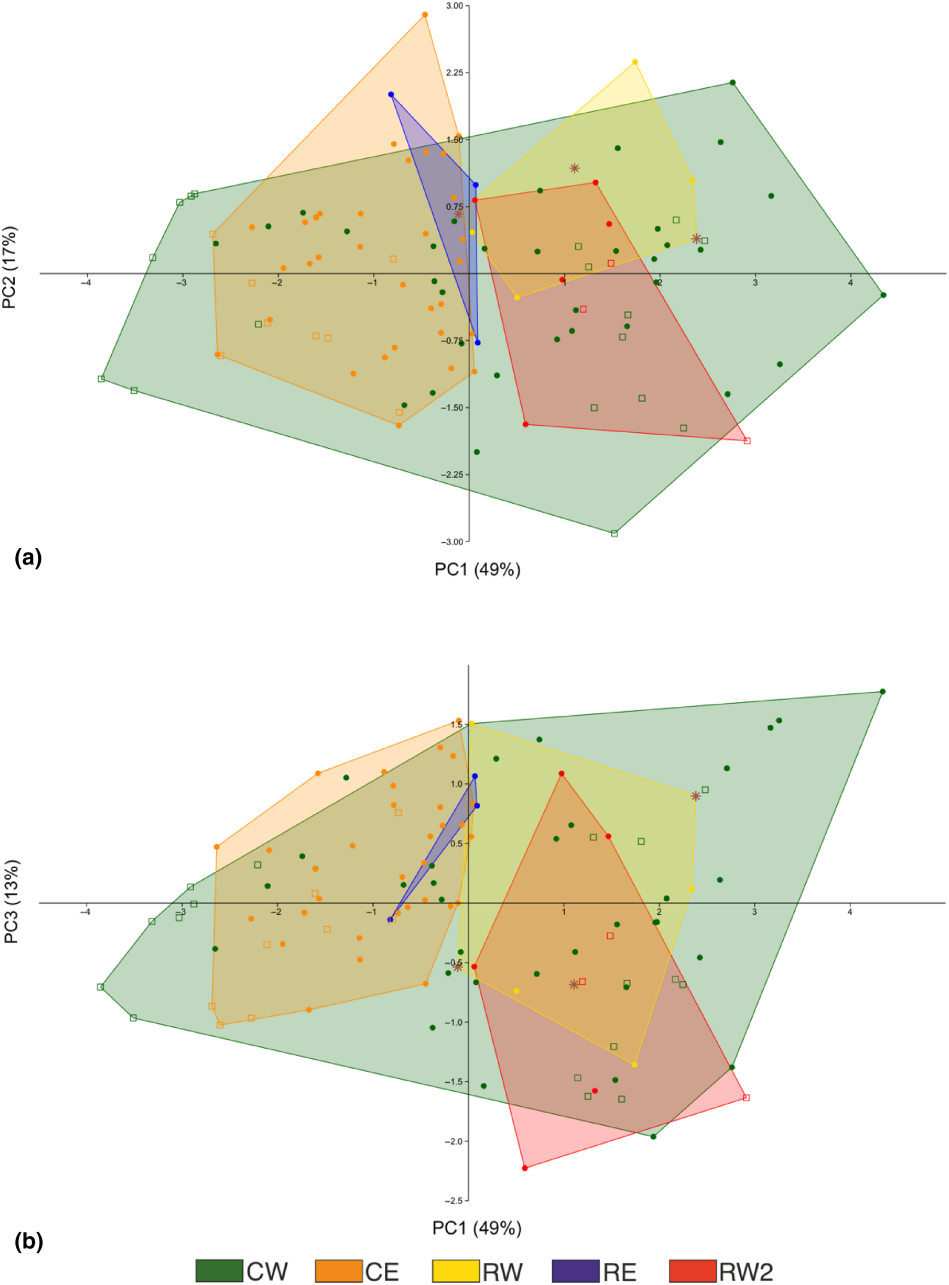
Principal component analysis of morphometric measurements. Scores of (a) PC2 and (b) PC3 are plotted against PC1 on the horizontal axis. The three components account for 49%, 17% and 13% of the variation in the analysis. The polygons enclose observations of each of the mitochondrial lineages, with colours explained in the legend. Specimens from stone habitats are presented by dots and those from sand by open squares. The specimens that had a contradictory CW versus RW lineage identity in COI versus ITS1 gene are shown as brown stars.

In the PCA of six morphometric variables, the first three components accounted for 49%, 17% and 13% of the total variance respectively (Figure 7). PC1 was positively correlated with most variables, at best with all the length measurements (*r* = .75–.8), and is interpreted as a general size component. PC2 was associated with widths of caudal rami and P5 endopod (*r* = .75 and .54), and PC3 with widths of P5 endopod and exopod (*r* = .56, .48) (Table S4 in Data S1). The ordination plots showed almost full separation of the Eastern CE and RE lineages from the Western RW and RW2 along the size axis PC1, RE appearing as a borderline case (Figure 7). The common CW lineage overlapped with all others covering the whole range of the PCI axis, whereas the means of CW versus CE (and vs. most others) were different. The specimens which had a contradictory CW versus RW lineage identity in COI versus ITS1 gene markers grouped within the cloud of RW lineage, which represents their nuclear background. Notably, the analysis showed no association between the lineage identity and the PC2 and PC3 axes, related to appendage widths and thus interpreted as shape components. There was no clear division of specimens collected from different substrates, although on the PC1 (size) axis the lowest scores were of specimens from sand and the highest ones from stone substrates.

## 4 DISCUSSION

### 4.1 Phylogenetic divergence and systematic subdivision

We describe a number of deep subdivisions within *Harpacticella inopinata*, one of the most peculiar endemic harpacticoid copepods of Lake Baikal, distributed all around the coasts of the lake. We identify five main units, or putative taxa, which are principally characterized by distinct and deep mitochondrial lineages, but also by a concurring subdivision in nuclear markers of the rRNA operon, that is, 28S and ITS, and by a set of morphological phenotypic differences which are largely congruent with the molecular subdivision but are distributed in a mosaic pattern among taxa. These five units show features of both allopatric and sympatric geographical distribution and may be divided into two main groups: a pair of widely distributed but largely allopatric units (CE and CW), and a set of rare units (RW, RW2, RE) with more scattered distribution. While the presence of the distinct genetic lineages is verified by all three molecular markers, the complex pattern of systematic heterogeneity calls for evaluation of the taxonomic significance (and rank) of the subdivisions, and explanation of their evolutionary history. At any rate, common arguments supporting the independent status of each lineage include the high genetic distances from all other four (20%–35% in COI, 1.5%–3% in 28S), and their emergence as separate MOTUs in all delimitation analyses (Figure 2).

While all divergences were strong, the two common lineages CW and CE concordantly appeared as sister clades both in mitochondrial and 28S phylogenies. In qualitative morphological characters, they were generally similar to each other but showed differences from the rare lineages RE and RW in the Antenna II exopod (5–6 ‘crown‐like’ spinules on the tip of the Antenna II exopod) and P1 endopod setation (2 additional rudimental claw‐like spines at the tip of the P1 endopod; Figure 6). Unlike in qualitative morphology, the CE and CW lineages however differed statistically in the means of almost all morphometric measurements (Figure S9 in Data S2). Yet, there was great overlap in the measurements and the difference seemed to be largely about the narrower range of observed variation in CE (Figure 7; see further below). These two common lineages were mainly allopatrically distributed along the Eastern and Western shores, with a few cases of sympatry in their contact zones.

The rare RE and RW lineages in turn appeared as sister clades in mtDNA phylogeny (Figure 2a), while that signal was not as clear as for CW versus CE and was not repeated in nuclear 28S (Figure 3a). Morphologically they differed from the C lineages by having only two to three ‘hair‐like’ spinules on the tip of the Antenna II exopod, and just four claw‐like spines at the tip of the P1 endopod (Figure 6). Morphometrically, they differed in P5 exopod width. Rare lineages mainly occurred in sympatry with each other or with the common lineages.

The RW2 lineage was similarly distinguished by molecular characters but had no clear sister lineage either in molecules or morphology. Rather it possessed a unique combination of morphological character states, sharing the state of CW + CE in P1 claw‐like spines but that of RW + RE in A2 setation. Morphometrically, RW2 was grouped with other western lineages CW and RW, and stood out with its longest P5 exopods and endopods (Figure S9 in Data S2). RW2 was mostly found in sympatry with other lineages and only in the Southern basin. A problem is the small number of individuals studied, but qualitatively the same arguments for independent history should apply as for the two composite groups above.

In a parallel study of *H. inopinata* on a local scale in Southern Baikal (the Listvyanka and Bolshie Koty sites), Fefilova et al. (2023) also recorded both molecular and morphological differences, in a character set overlapping ours (*COI*, A2 exopod and P1 endopod). They also recognized three deep molecular lineages at these sites but, unlike this study, did not directly connect those to the morphological differences, as different specimens were used for the molecular and morphological analyses.

Our judgement of the systematic significance of the observed variation relies on the general molecular divergence, the congruence of mitochondrial and nuclear differences, the congruence between molecular and morphological character differences, reproductive isolation inferred from sympatric occurrences, and evidence of stable subdivision seen both in areas of allopatry and of overlapping distributions. The data strongly point to the long independent histories and predicted independent future of the main lineages, justifying the hypothesis of full species. This interpretation is not overturned by the evidence of limited introgression or character incongruence (see below) which are now seen as common phenomena among species in nature. The geographical distributions also do not fit an alternative hypothesis of a subspecific rank that relies on a parapatric pattern in multicharacter differences, as elaborated for ancient lake taxa in Daneliya and Väinölä (2014). While, unlike for other pairs of taxa, we do not as yet have a morphological diagnosis to separate the two most common ones CE and CW, the overall evidence for their distinction and independence is not essentially weaker than for the others, such as to justify a different taxonomic interpretation. For the present, we will refer to the five proposed species with the temporary informal names *Harpacticella inopinata* CE, *H. inopinata* CW, *H. inopinata* RW, *H. inopinata* RE and *H. inopinata* RW2.

### 4.2 Cases of mitochondrial versus nuclear discordance

Apart from the general concordance of the individual COI and nuclear ITS lineage identities, there were a number of instances of discordance. Individual specimens from three localities in the northern basin were represented by a CW lineage mitotype but an RW type ITS1 sequence and also had a (predominantly) RW morphological phenotype (Figures 1d and 3d; Figure S8 in Data S2). Similarly, two specimens from the southern Listvyanka were of a CW mitotype but RW2 ITS1 phenotype (Figure 2a; Figure S3 in Data S2). While the discordance could be seen as a challenge to the species hypothesis, it more plausibly reflects the general proneness of mtDNA to introgression, following rare events of hybridization between even well‐defined species (e.g. Cristescu et al., 2010; Nevado et al., 2009). In these instances, we identify the individuals by their nuclear phenotype (ITS and/or morphology), although they bear an ‘alien’ introgressed mitochondrion. This interpretation fits well with the inferred demographic histories of the taxa (see below): the CW mitochondrion is inferred to have spread through the lake comparatively recently, occupying regions where the rare species would have resided for longer times. If such a spread occurred by a mitochondrial selective sweep, an interspecific transfer would actually appear plausible. Comparable cases of introgression between sister lineages of resident populations and recurrent colonists have been reported from multilocus studies in other lacustrine species flocks, for example, from diaptomid copepods from Sulawesi (Vaillant et al., 2013), cichlid fishes of the East African Lakes Victoria, Tanganyika and Malawi (Nevado et al., 2009; Salzburger et al., 2002; Won et al., 2005), and the cichlids in crater lakes of Cameroon and Nicaragua (Barluenga et al., 2006; Schliewen & Klee, 2004).

### 4.3 Comparative crustacean diversity in Lake Baikal

The finding of five separate cryptic species within one taxon adds a new viewpoint to the discussion of unique harpacticoid crustacean diversity in Lake Baikal (Okuneva, 1989; Timoshkin, 2001). The typically benthic harpacticoids are considered to have undergone extensive radiations due to the changes inf bottom landscapes and the formation of abyssal habitats during lake development (Martens & Schön, 1999). The exceptional morphological diversity is however restricted to the family Canthocamptidae with 77 known species and several subflocks of species. For example, the *Bryocamptus* and *Moraria* flocks include 23 and 25 widespread species within the lake respectively. However, the relatively small morphological differences within the genera point to a relatively young age of these flocks (Okuneva, 1989). The conventional harpacticoid diversity is both sympatric (e.g. 47 species recorded in Bolshie Koty alone) and allopatric, with individual species restricted to each of the main Northern, Central or Southern sub‐basins. *Harpacticella inopinata* is the single representative of Harpacticidae, and as such overall the most widespread species in the littoral zone (Timoshkin, 2001). The new subdivision is not completely unexpected, however; Evstigneeva and Sobakina (2008) already speculated on a subdivision based on variation in setation of Antenna II and of P1 legs, that is, the same characters that separate the common and rare species groups in this study.

While Baikalian harpacticoids have previously been studied only morphologically, integrative morphological and molecular approaches leading to recognition of new taxa and complexes of cryptic species have been undertaken in other crustaceans, including amphipods (Daneliya & Väinölä, 2014; Gurkov et al., 2019), ostracods (Schön et al., 2017) and cyclopoid copepods (Mayor et al., 2017). Our data similarly revealed a complex of cryptic taxa within conventional species. Nevertheless, the depth of the interlineage divergence appears exceptional in *Harpacticella* (up to 37%/18% in model‐corrected and uncorrected p‐distances). For instance, in amphipods, uncorrected distances ranged from 3% to 11% between cryptic species and reached up to 20% between morphological species in one species flock (Daneliya & Väinölä, 2014; Gurkov et al., 2019). In ostracods, model‐corrected distances of c. 25% between cryptic species were reported (Schön et al., 2017).

### 4.4 Patterns of geographical distribution

Both allopatric and sympatric patterns of distribution were observed among the new *Harpacticella* species. In a broader Baikalian framework, an allopatric or parapatric distribution of closely related species or lineages appears to be the more common pattern, especially in benthic species, where microgeographical and intralacustrine isolation might play an important role. Thus, the cryptic subdivision of *Eulimnogammarus* amphipods was among distinct Eastern, Western and Southern lineages (Gurkov et al., 2019). Likewise, the *Dorogostaiskia parasitica* complex comprised five geographical subspecies along the Eastern and Western shores (Daneliya & Väinölä, 2014). In *Harpacticella* the two common species showed a mainly allopatric pattern along Eastern and Western shores, but with several exceptions. Sympatric occurrences in the central part of the lake were found both on the Western side on Olkhon Island and on the eastern shore at Baklanyi, and *H. inopinata* CW also occurred near the eastern shore on Malyi Ushkanii island. The Uskanii islands however represent the extreme point of the mainly submerged Academician Ridge which extends from the Olkhon island and separates the Central and North Baikal sub‐basins. This ridge was probably above the water surface until the Late Pleistocene (0.1–0.15 Ma) and therefore could have provided continuous littoral habitat that enabled spread from the west (Mats et al., 2000). In a parallel manner, the Western haplogroup of the amphipod *E. verrucosus* is present on the Bolshoi Ushkanii Island (Gurkov et al., 2019). Another uplift, the Selenga‐Buguldeika ridge, submerged some 0.2 Ma ago, is situated near the Baklanyi site (Logatchev, 1993; Mats et al., 2011). The two species *H. inopinata* CE and CW also overlap in their contact in the southern end of the lake.

A basic East–West allopatric separation was also observed among *H. inopinata* RW, RW2 and RE (Figures 2d and 3d). At the same time, these three rare species occurred sympatrically, either with the common or with the other rare ones. Sympatry of deep conspecific lineages or morphological species has been previously reported in Lake Baikal and is mainly attributed to ecological specializations to types of substrate (Schön et al., 2017), depths (Timoshkin, 2001) and feeding modes (Kovalenkova et al., 2020). While sympatric occurrences of common and rare taxa of *H. inopinata* serve as an additional argument for their species status in terms of reproductive isolation, we cannot currently assess which factors underlie these distributions. Interestingly, the sympatric occurrences were found in contact areas of the circular surface currents (Figures 2d and 3d), suggesting that the currents are involved as a dispersal factor. Nevertheless, there is no indication the observations of sympatry would be explained by the type of substrate, and more detailed environmental analyses would be needed to investigate the presence of specializations in *H. inopinata*.

### 4.5 Rates and dates of diversification

Interpreting the patterns of variation in an evolutionary framework depends on setting them in the temporal context of the geological and climatic history of the lake, and this entails implicating a molecular rate. The time scale of the emergence of the hyperdiverse Baikalian biota in itself is a long‐debated issue (Mats et al., 2011; Timoshkin, 2001). The evolution of the rift lake basin through the Oligocene‐Miocene (protobaikalian stage) took place in very different climatic settings from the present. The current neobaikalian lake evolved with the climatic cooling in the Late Pliocene‐Pleistocene (~3 Ma), and finally was affected by the glacial cycles that would have repeatedly shifted the geographical zones ecologically favourable to given species (Kovalenkova et al., 2020; Pudovkina et al., 2014). Molecular estimates have led to mixed conclusions of the age of the current diversity in different groups of taxa (e.g. Hausdorf et al., 2003; Maikova et al., 2015; Papousheva et al., 2003), and the discoveries of a new level of abundant cryptic diversity besides the recognized morphological diversity brings another level of complexity to be explained.

Specifically, interpreting harpacticoid diversity is complicated by a lack of any copepod fossil record that would enable ‘direct’ calibration of molecular rates, and attempts for biogeography‐based calibration are also rare (Selden et al., 2010). As a basic reference, we therefore use ‘a general crustacean rate’ 2.6% Ma^−1^, an average from a set of COI studies from Copepoda and Malacostraca taxa (see Section 2). However, applying these rates to the diversification and spread of the *H. inopinata* complex yields dates that are inconveniently old in the framework of lake history. This is not a unique situation, but was also repeatedly encountered in interpreting the origin of cryptic diversity in Baikalian amphipods (Daneliya et al., 2011; Gurkov et al., 2019; Romanova et al., 2016). A molecular rate even five times higher was therefore suggested for these crustaceans (Naumenko et al., 2017). For adjusting the discussion of *Harpacticella* we also considered such a fivefold rate in parallel (13% Ma^−1^) (Table 2, Figure 4).

### 4.6 Demographic histories

The patterns in mitochondrial haplotype variation, interpreted as reflecting population size history, varied clearly among the five taxa (Figures 1b and 3; Table 2). The two common ones *H. inopinata* CE and *H. inopinata* CW both showed shallow genealogies and star‐like haplotype networks, patterns expected from a relatively rapid demographic expansion following a bottleneck phase (Maturana et al., 2022). It seems that the current populations of the two species, or at least their mitochondria, each trace back to a (local) ancestral population, from which their genes have spread through their current ranges ‘relatively recently’. It is not necessarily clear whether or not the demographic and geographical expansions would have been simultaneous (expansion along with spread vs. expansion followed by spread). Notably, however, there seems to be no clear geographical structure in the variation within these species in the lake. The traces of demographic history only extend to the intra‐lineage coalescences, that is, to the inferred expansion ages, which in these cases are far younger than the inter‐lineage divergence (in terms of distance, 2% vs. 35%). The intralineage data are then unlikely to be informative of the phenomena related to species diversification itself, and we cannot speculate on the lineage distributions from times pre‐dating the expansion (or even during that).

At the same time, the rare species *H. inopinata* RE and *H. inopinata* RW show localized distributions but deeper genealogies (up to 7%), and no evident signals of recent expansions. Still, there is geographical structure, and different populations making separate branches in the mitochondrial trees. This structure could reflect relatively stable populations in the long run. In the temporal perspective of lake history on the one hand and with the contrasting signal from the widespread species on the other, such stability may however appear counter‐intuitive, if the structure indeed would have persisted since times before the spread of common lineages. The limited distribution and possibly long persistence of the rare taxa might suggest that they experienced population declines, which however was not reflected in our analyses. Another suggestion is that rare species could represent an older Baikalian fauna that inhabited the lake before the formation of ultradeeps (cf. Okuneva, 1989).

With the common molecular rates, the inferred demographic expansions in *H. inopinata* CE and *H. inopinata* CW would date back 0.8 to 0.3 Ma, that is, several major glacial cycles ago, and even with the fivefold rates from 0.15 to 0.07 Ma, close to the last major interglacial (Table 2). The current homogeneous populations could then reflect either survival or maintenance of the newly spread interglacial populations in their broad distributions through the environmental perturbations of the last glacial maximum (LGM, ~20 ka), or locally restricted survival of very large populations and their post‐glacial spread. Even these scenarios do not appear particularly plausible, and we would rather consider a hypothesis of even faster mutation rates with which the expansion signal would have arisen only in the post‐glacial time, with spread facilitated by the warming of the climate. Such rates have been suggested in some other post‐glacial crustacean populations (Audzijonyte & Väinölä, 2006; Loehr et al., 2023). They are also related to the general observation of ‘apparent acceleration of molecular rates’ in recent populations (e.g. Crandall et al., 2012; Maturana et al., 2022). Whatever the time scale and rates have been, a difference between the two widespread species that deserves attention is that the expansion peak appears two times older in *H. inopinata* CE than *H. inopinata* CW, and if rates were the same this would imply a clearly earlier spread along the eastern shore.

### 4.7 Age of cryptic radiation

As regards the age of *H. inopinata* lineage divergence, which conventionally would be equated with speciation, the common rates around 2.6% Ma^−1^ would place it in the Miocene 6–23 Ma ago (Figure S5 in Data S2). Even older divergence (up to 41 Ma, Figure S6 in Data S2), approaching the age of Lake Baikal itself, and lower molecular rates ~1.5% Ma^−1^, would be inferred if applying (secondary) calibration ages inferred for splits between Copepoda orders from a broader phylogenomic analysis (Eyun, 2017). In the framework of the lake history, a retention of practically cryptic diversity from times when the environment (and consequently species ecology) were drastically different from the present world does not make sense; it is hard to figure out how even the conventional morphological diversity of Baikalian organisms could have been conceived in such unfamiliar settings (cf. Mats et al., 2011; Väinölä & Kamaltynov, 1999). Nevertheless, similar ages (20–26 Ma) have been suggested, for instance, in the *Sergentia* chironomid species flock (Papousheva et al., 2003) and *Diacyclops* copepods (Mayor et al., 2017) based on the conventional molecular rates.

With the proposed higher rate, *H. inopinata* would in turn have radiated some 2–4 Ma ago (Figure S5 in Data S2), which matches the beginning of neobaikalian stage, climatic cooling and evolution of the present type of lake environment. The emergence of new environments, including the origin of oxygenated deep‐water zone has often been linked with the morphological and ecological radiations of the current fauna, and molecular data from some taxa, including fishes, also match this idea even when judged with conventional rates (e.g. Kontula et al., 2003; Maikova et al., 2015). More commonly this period is now associated with cryptic radiations, below the conventional species level (e.g. Bukin et al., 2018; Gurkov et al., 2019; Schön et al., 2017; Zaidykov et al., 2020), which would leave the morphological diversification to earlier times and environments.

In harpacticoids, the judgement is complicated by the fact that *H. inopinata* sensu lato represents the single conventional species of its family in Lake Baikal, with no evidence of earlier morphological radiation. At the same time, radiation with tens of endemic species is recognized in another harpacticoid family Canthocamptidae, but as there is so far no molecular characterization of that diversity we cannot set the newly recognized taxa on a scale of conventional Baikalian species diversity. On the other hand, records of cryptic diversity abound in copepods of other lake and marine environments, and the distances between those ‘conspecific’ lineages are often similar and even larger than those in *Harpacticella*, up to 70% (Baek et al., 2016; Bruno et al., 2017; Figueroa et al., 2020; Garlitska et al., 2012; Karanovic & Cooper, 2012; Kochanova et al., 2021; Marrone et al., 2013; Previšić et al., 2016; Thum & Harrison, 2009). In marine Harpacticidae, distinct cryptic species with more than 20% COI divergence have been recorded in *Zausodes arenaceous* (Easton et al., 2010), *Tigriopus californicus* (Willett & Ladner, 2009) and *T. fulvus* (Vecchioni et al., 2019). A higher molecular rate has been suspected to characterize this group in general (Barreto et al., 2018). With all this background, it is evident that reference to the conventional crustacean rates will not serve to understand the time scale of diversification in Baikalian *Harpacticella*.

### 4.8 Morphometric variation related to molecular lineages and habitat type

While the morphometric data also showed significant differentiation both among the molecular lineages and between types of habitat, their use in practical taxonomy remains problematic—in contrast to the qualitative morphological traits. Overall, the metric differences among lineages were mainly related to general size (represented by PC1, Figure 7) rather than to shape, and then showed a prominent geographical east–west component that does not concur with the tree of mitochondrial relationships. On the other hand, there were differences between the habitat types in three of the six measurements, which were larger on stone than on sand bottoms (Figure S9 in Data S2). The habitat type was not clearly connected to lineage identity, however (Table 1, Figure 1). Although significant, the morphometric differences between East and West were subtle and difficult to identify without actual measurement, for example, from microscopic photographs. The variation of appendage forms and shapes was seen even at an intrapopulation level, which indeed is a typical phenomenon in copepods, and is often pointed out in identification guides (Borutzky, 1952). Such variation is well illustrated in Figure 5b,c with two individuals of *H. inopinata* CE that have alternatively the short and elongated forms of P5 exopod, and two of *H. inopinata* CW with short and elongated caudal rami respectively. Considerable intraspecific variability of the same morphometric characters has repeatedly been reported in other copepods. It has variably been interpreted in terms of new systematic divisions (Lajus et al., 2015; Lajus & Alekseev, 2000) or just as characteristically high intrapopulation variation or teratology (Borutzky, 1952). At this point, we refrain from including the morphometric variations in the characterization of the new systematic subdivision of *H. inopinata* sensu lato.

## 5 CONCLUSIONS

In an analysis of molecular and morphological variation, the endemic harpacticoid crustacean *Harpacticella inopinata* sensu lato, widespread within Lake Baikal, was found to comprise a complex of five cryptic species, at least. Two sister taxa, *H. inopinata* CE and *H. inopinata* CW, have broad but largely allopatric distributions and appear to have spread relatively late through their ranges, while three other species have more scattered distributions but a longer molecular record of demographic history. Considering the deep molecular divergence, inferred demographic histories and the geological evolution of the lake, the rate of molecular evolution in the Baikalian *H. inopinata* complex seems to have been faster than that of crustaceans in general. While genetically all five species show congruent deep divergence in all molecular markers, qualitative morphological characters only distinguish the part of them, and quantitative morphometry indicates a geographical east–west component between species.

## AUTHOR CONTRIBUTIONS

**Elena Kochanova:** Conceptualization (equal); data curation (equal); formal analysis (lead); investigation (lead); visualization (lead); writing – original draft (lead); writing – review and editing (equal). **Tatyana Mayor:** Data curation (equal); investigation (supporting); writing – review and editing (supporting). **Risto Väinölä:** Conceptualization (equal); formal analysis (supporting); supervision (lead); writing – review and editing (equal).

## CONFLICT OF INTEREST STATEMENT

The authors declare no conflict of interest.

## Supporting information


Data S1:

Data S2:

## Data Availability

The COI, 28S and ITS1 gene sequences obtained in this study are available in GenBank under accession numbers OR506752–OR506893, OR509834–OR509843 and OR826812–OR826816 respectively. The alignment of ITS1 sequences was uploaded to the Dryad repository (http://doi.org/10.5061/dryad.qrfj6q5nr). The museum vouchers IDs, and primary morphological, morphometric and haplotype frequency data are in the Electronic supplementary materials.
