# Supplementary material for: Cryptic diversity and speciation in an endemic copepod crustacean Harpacticella inopinata within Lake Baikal
Source: Ecol Evol. 2024 May 31;14(6):e11471. doi: 10.1002/ece3.11471 (PMC11140236; doi:10.1002/ece3.11471)
Supplement: Supplementary file 2 — Data S2: [file ECE3-14-e11471-s002.docx]

Kochanova, Mayor & Väinölä (2024): Cryptic diversity and speciation in an endemic harpacticoid copepod crustacean *Harpacticella inopinata* within Lake Baikal. Ecology and Evolution.

**ESM2:**

**Tables S1-S2**

**Figures S1-S13**

Table S1. Primers pairs and thermal cycling protocols for the three sequenced genes. Each cycling protocol is preceded by 4 min at 95 °C, and followed by 7 min at 72 °C.

| Locus, primers and primer reference | Primer (5’-3’) | Thermal cycling protocol |
| --- | --- | --- |
| COI  L1384-COI:  Machida et al., 2004;  HC02198:  Folmer, 1994 | GGT CAT GTA ATC ATA AAG ATA TTG G  TAA ACT TCA GGG TGA CCA AAA AAT CA | [30 s at 95 °C, 30s at 50 °C, 50 s at 72 °C] x 35 |
| COI  Hin_13F:  Hin_630R:  Present study | CCT GAG TGT TCT DAT YCG GC  GTG GAG TTC AGG TTA CGG TC | [30 s at 95 °C, 30s at 50 °C, 50 s at 72 °C] x 35 |
| ITS1  SP-1-5’:  SP-1-3’:  Chu et al., 2001 | CAC ACC GCC CGT CGC TAC TA  ATT TAG CTG CGG TCT TCA TC | [30 s at 95 °C, 30s at 50 °C, 70 s at 72 °C] x 38 |
| 28S  28S-F1a:  28S-R1a:  Ortman, 2008 | GCG GAG GAA AAG AAA CTA AC  GCA TAG TTT CAC CAT CTT TCG GG | [60 s at 95 °C, 60s at 50 °C, 60 s at 72 °C] x 35 |

Machida, R. J., Miya, M. U., Nishida, M., & Nishida, S. (2004). Large-scale gene rearrangements in the mitochondrial genomes of two calanoid copepods Eucalanus bungii and Neocalanus cristatus (Crustacea), with notes on new versatile primers for the srRNA and COI genes. *Gene*, *332*(1–2), 71–78. https://doi.org/10.1016/j.gene.2004.01.019

Folmer, O., M. Black, W. Hoeh, R. Lutz, and R. Vriyenhoek. 1994. DNA primers for amplification of mitochondrial cytochrome c oxidase subunit I from diverse metazoan invertebrates. Mol. Mar. Biol. Biotech., 3:294–299.

Chu, K. H., Li, C. P., & Ho, Y. (2001). The first internal transcribed spacer (ITS-1) of ribosomal DNA as a molecular marker for phylogenetic and population analyses in Crustacea. Marine Biotechnology, 3: 355–361.

Ortman B.D., 2008. DNA Barcoding the Medusozoa and Ctenophora. University of Connecticut; Storrs.

Table S2. Mean model-corrected distances (below diagonal) and observed p distances (above diagonal, %) between *H. inopinata* taxa (MOTUs) identified by species delimitation methods from the 500-bp COI fragment sequences.

| Lineage | RE | RW | RW2 | CE | CW | 3Kt | 4Kt | 93Slyud | 94Slyud | 117Tank |
| --- | --- | --- | --- | --- | --- | --- | --- | --- | --- | --- |
| RE | * | 11.5 | 15.1 | 18.1 | 17.9 | 13.7 | 16.7 | 14.8 | 16.7 | 17.6 |
| RW | 20.2 | * | 16.4 | 18.3 | 16.6 | 6.2 | 16.5 | 13.8 | 18.4 | 19.4 |
| RW2 | 29.0 | 30.3 | * | 17.9 | 16.6 | 18.4 | 17.3 | 16.6 | 6.7 | 17.8 |
| CE | 36.5 | 37.2 | 34.2 | * | 18.1 | 21.1 | 19.4 | 9.4 | 19.5 | 10.9 |
| CW | 35.6 | 32.6 | 30.3 | 34.7 | * | 17.3 | 15.9 | 9.4 | 17.5 | 8.0 |
| 3Kt | 23.7 | 7.7 | 32.0 | 39.1 | 32.4 | * | 17.0 | 15.2 | 18.4 | 20.2 |
| 4Kt | 32.5 | 30.1 | 31.3 | 37.7 | 26.8 | 30.2 | * | 17.0 | 17.4 | 18.2 |
| 93Slyud | 25.7 | 22.9 | 28.7 | 13.0 | 12.8 | 25.6 | 30.4 | * | 17.4 | 10.0 |
| 94Slyud | 31.4 | 33.5 | 7.9 | 38.1 | 31.8 | 31.2 | 31.6 | 30.2 | * | 19.4 |
| 117Tank | 34.2 | 38.0 | 31.7 | 10.1 | 15.7 | 39.1 | 33.1 | 13.8 | 36.5 | * |


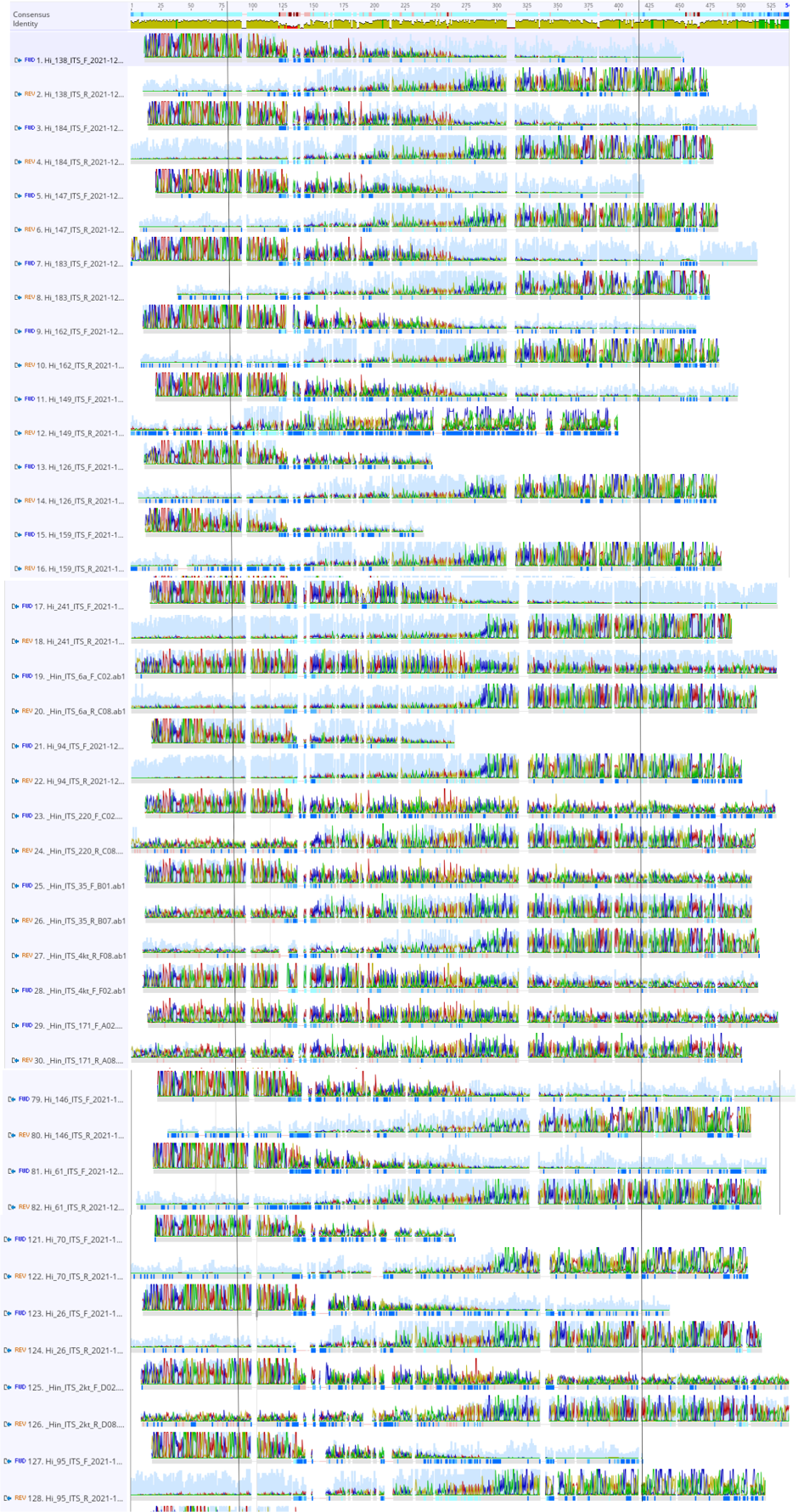


Figure S1. Picture of chromatograms of ITS1 and flanking regions of *Harpacticella inopinata*. Vertical lines indicate approximate start and end of the ITS1 alignment.


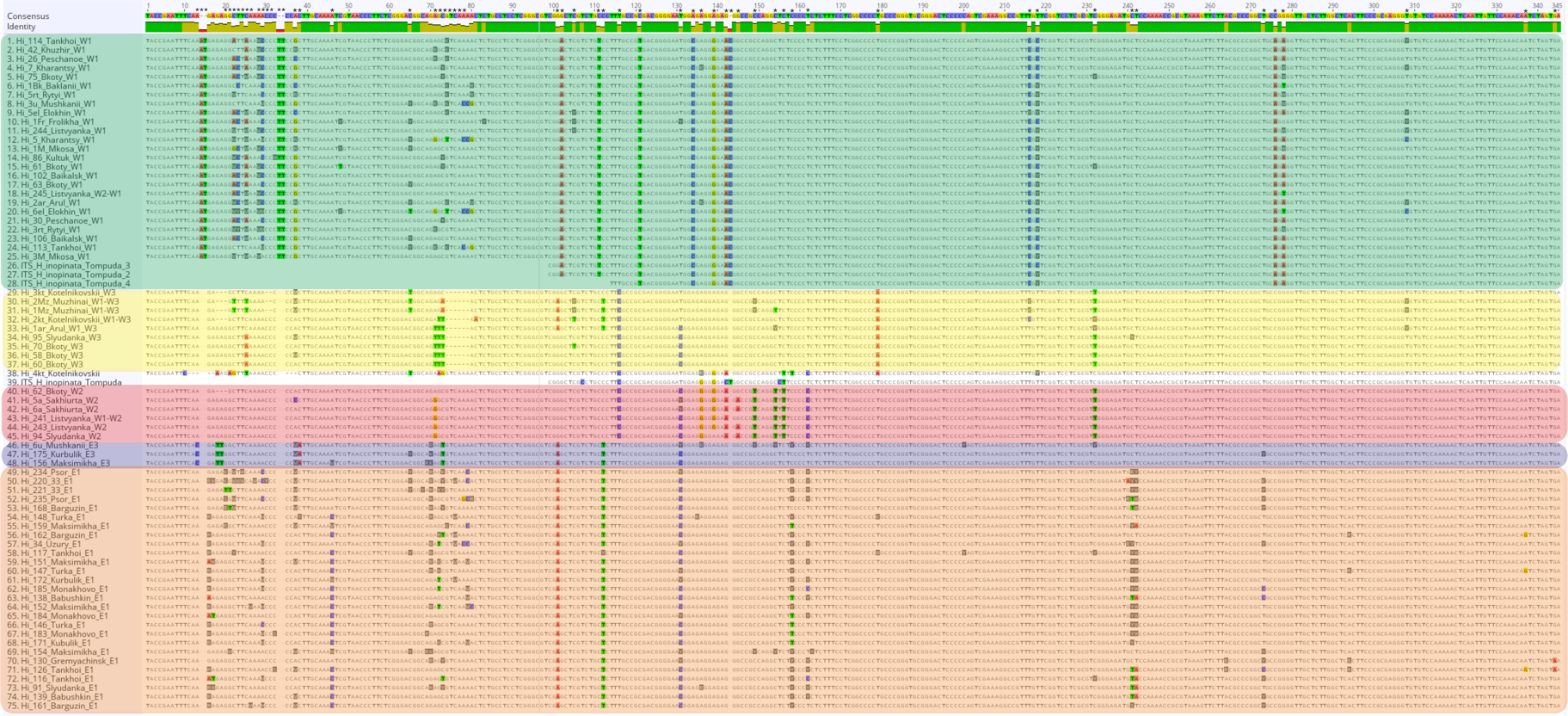


Figure S2. Parsimony informative characters (marked *) in the ITS1 dataset of *H. inopinata*. The main clades are marked by colours according to those in the tree diagrams.


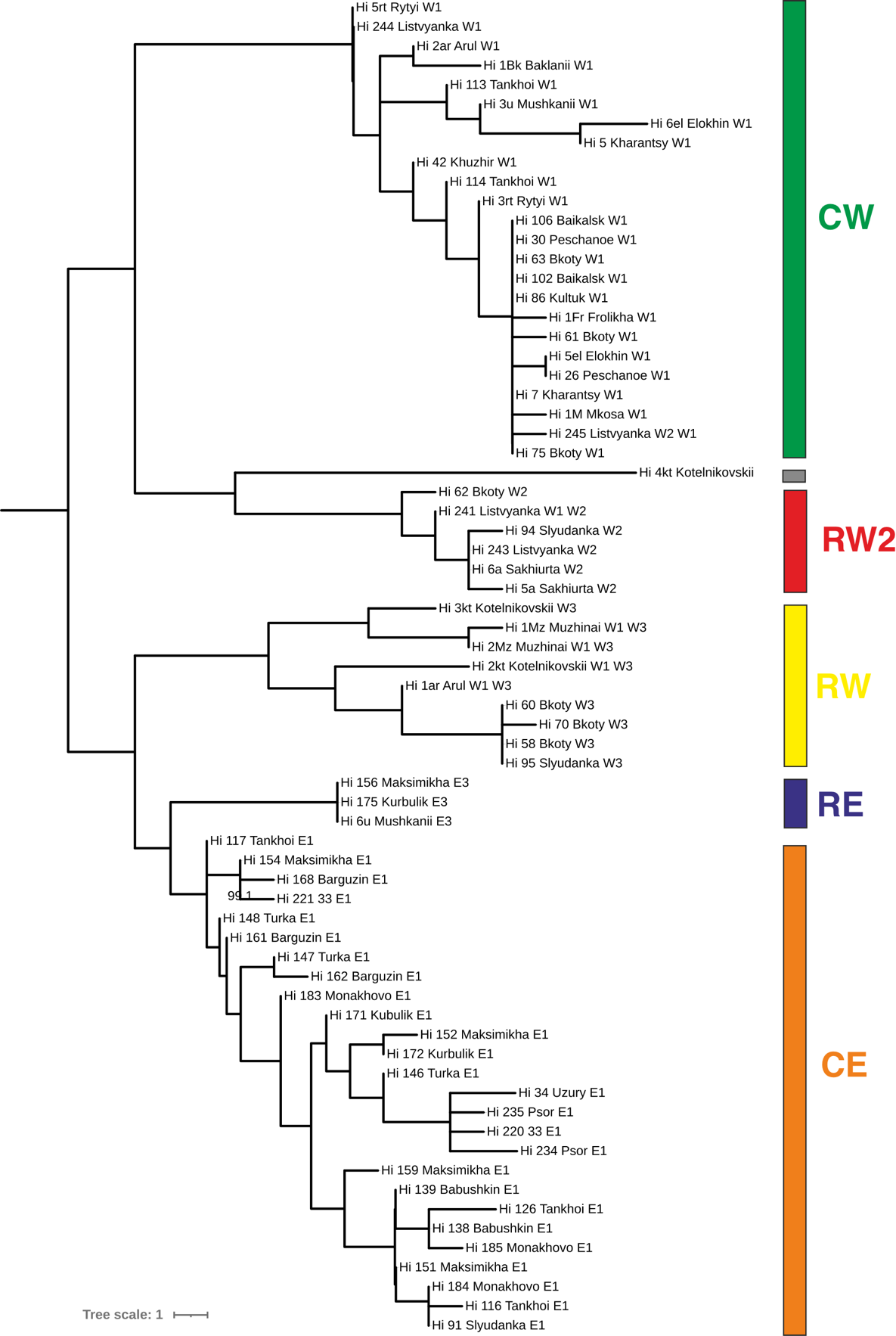


Figure S3. Maximum Parsimony tree from an alignment of all (gap-coded) ITS1 sequences.


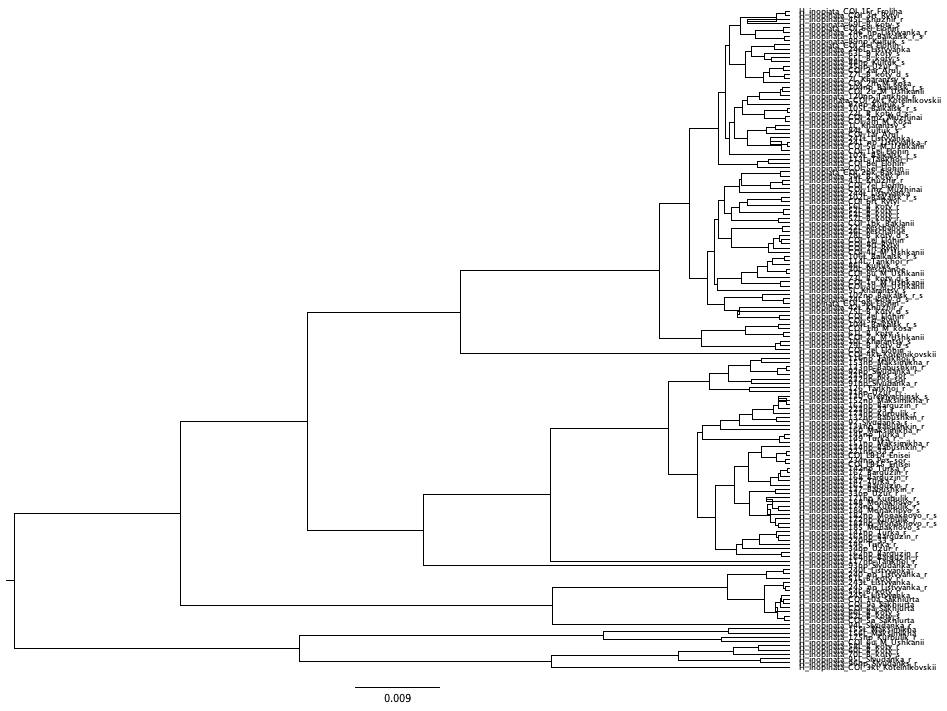


Figure S4. Bayesian tree from COI gene sequences for GMYC species delimitation analysis.


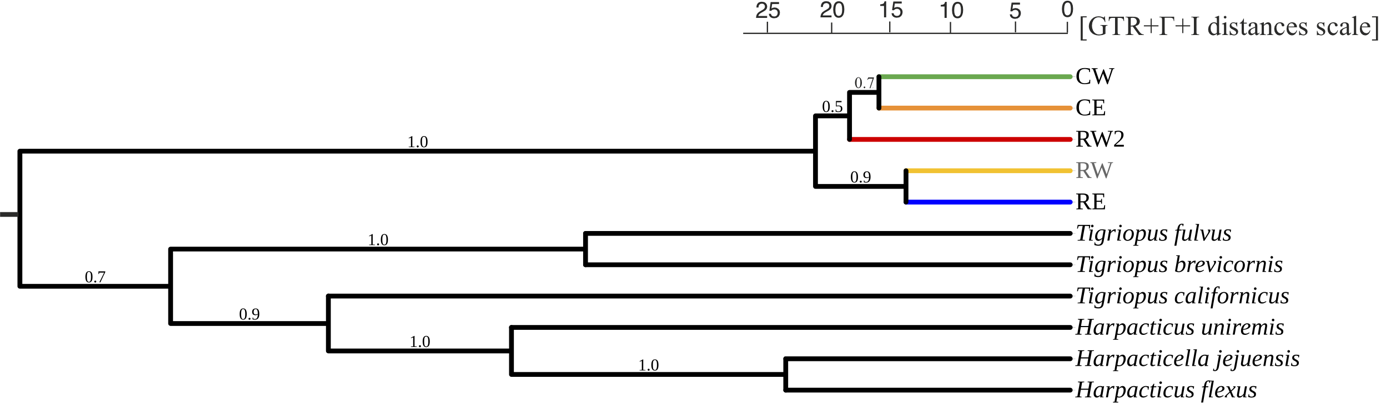


Figure S5. An ultrametric tree from COI sequence data of the five *Harpacticella inopinata* lineages and other Harpacticidae species, constructed in BEAST on the basis of GTR+I+Γ and Yule speciation tree prior model. The scale is related to the model-corrected distances (x 100) calculated for *H. inopinata*.


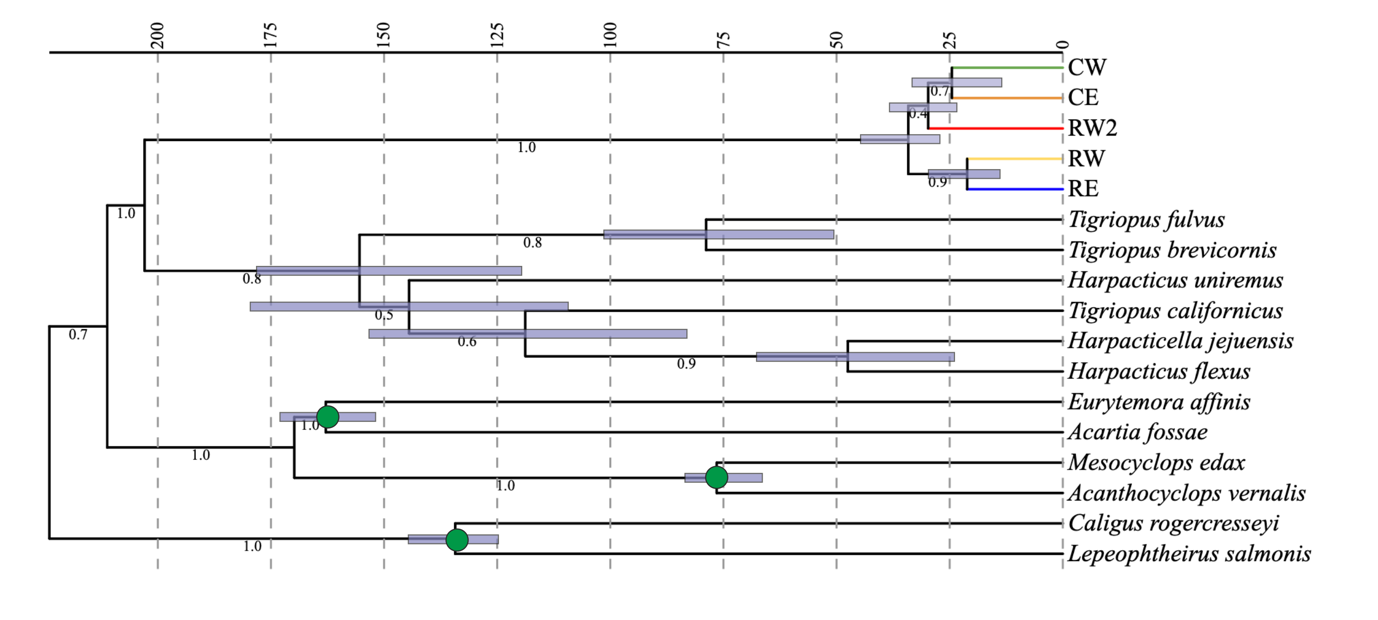


Figure S6. Time tree from COI gene data for *Harpacticella inopinata* lineages using the secondarily dated calibration nodes (green dots) from Euyn (2017). No priors on speciation rates were used. The harpacticoid outgroup is the same as in ML tree from COI data (Figure 1a). Numbers below the nodes are posterior probabilities. Highest probability density intervals (95% HPD) are shown for each node.

Method: To project the *H. inopinata* lineage relationships on an evolutionary time scale, a time calibrated species tree (chronogram) was created in BEAST2 (Bouckaert et al. 2014) using the COI gene dataset. While there are no reliable calibration points based on fossils within the harpacticoid order, we used dates for splits among external copepod taxa included as outgroups in the analysis. Three calibrating points obtained secondarily in broader phylogenomic analysis of copepod orders by Eyun (2017) were used: (1) 170 MYA as the split between the calanoids *Eurytemora affinis* (Poppe, 1880) and *Acartia fossae* Gurney, 1927, (2) 80 MYA between the cyclopoids *Mesocyclops edax* (Forbes S.A., 1891) and *Acanthocyclops vernalis* (Fischer, 1853), (3) 140 MYA is the divergence between siphonostomatoids *Caligus rogercressei* Boxshall & Bravo, 2000 and *Lepeoptherius salmonis* (Krøyer, 1837). The calibrated Yule model for the tree prior, the uncorrelated relaxed clock model proposed by Drummond et al. (2006), and the substitution model GTR+G(0.63)+I(0.32) from the ML analysis were used. The global substitution rates were estimated from calibration nodes, and no specific priors for speciation rates were applied since they might differ between Baikalian and non-Baikalian taxa (see Discussion). Posterior probabilities were determined from two independent MCMC runs of 20 million generations, using the selected mutation models, excluding the initial 25% of each run as burn-in. The stability of the runs and convergence of the MCMC were assessed with Tracer version 1.5 (http://beast.bio.ed.ac.uk/Tracer).

Result: The topology of the time-tree was the same as in the ML tree (Fig. 1a). From this analysis the splits between the five lineages would date back to the Miocene-Oligocene, 10 to 41 Ma, approaching the age of the Baikal itself. When the model-corrected distances (Fig. 1c) are compared with the branch lengths and divergence dating, the divergence rate would be estimated at ~1.5% Ma -1, which corresponds to the common rates observed in crustaceans (Marrone et al., 2013).

Bouckaert, R., Heled, J., Kühnert, D., Vaughan, T., & Wu, C.-H. (2014). BEAST 2: A Software Platform for Bayesian Evolutionary Analysis. *PLoS Comput Biol*, *10*(4), 1003537. <https://doi.org/10.1371/journal.pcbi.1003537>

Drummond, A. J., Suchard, M. A., Xie, D., and Rambaut, A. (2012). Bayesian phylogenetics with BEAUti and the BEAST 1.7. Mol. Biol. Evol. 29, 1969–1973. doi: 10.1093/molbev/mss075

Eyun, Si. (2017). Phylogenomic analysis of Copepoda (Arthropoda, Crustacea) reveals unexpected similarities with earlier proposed morphological phylogenies. BMC Evol Biol 17, 23. <https://doi.org/10.1186/s12862-017-0883-5>


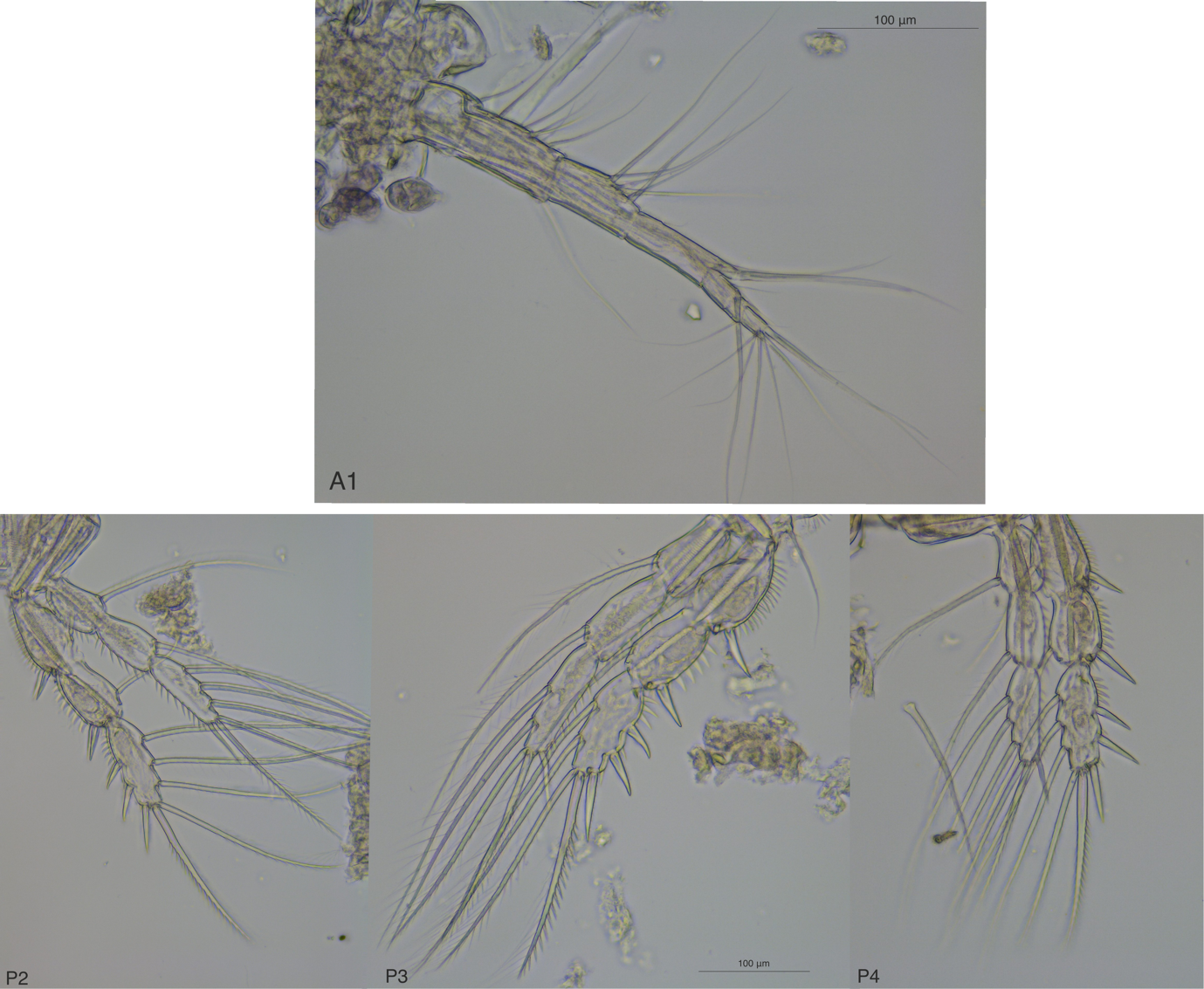


Figure S7. Photographs of Antenna I (A1), and second (P2), third (P3) and fourth (P4) swimming legs of *Harpacticella inopinata* from CW lineage (specimen 7 from Kharantsy).


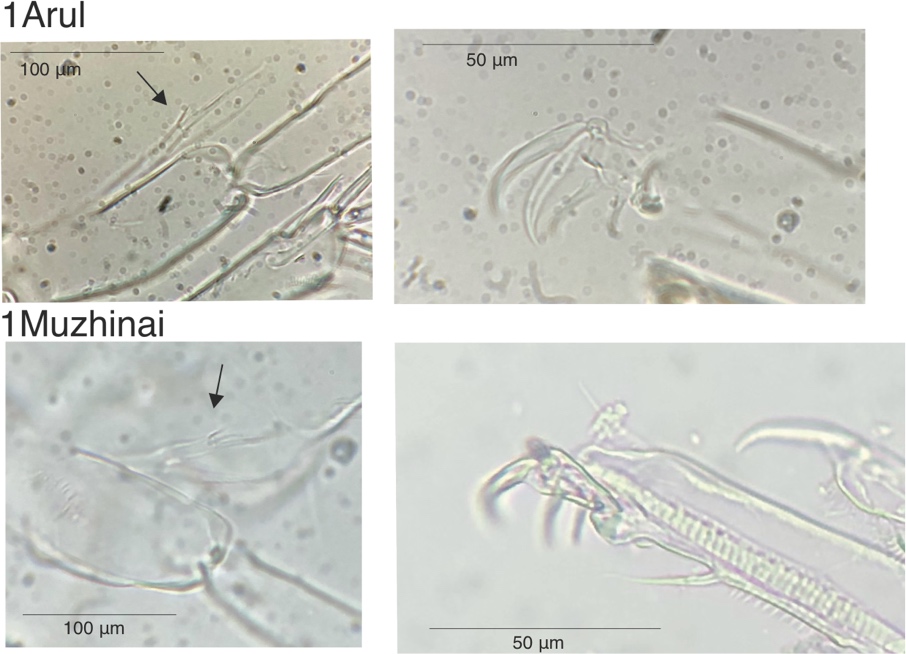


Figure S8. Photographs of Antenna II exopods (left) and P1 claws (right) of specimens 1Ar (from Arul) and 1Mz (from Muzhinai) of *Harpacticella inopinata*.


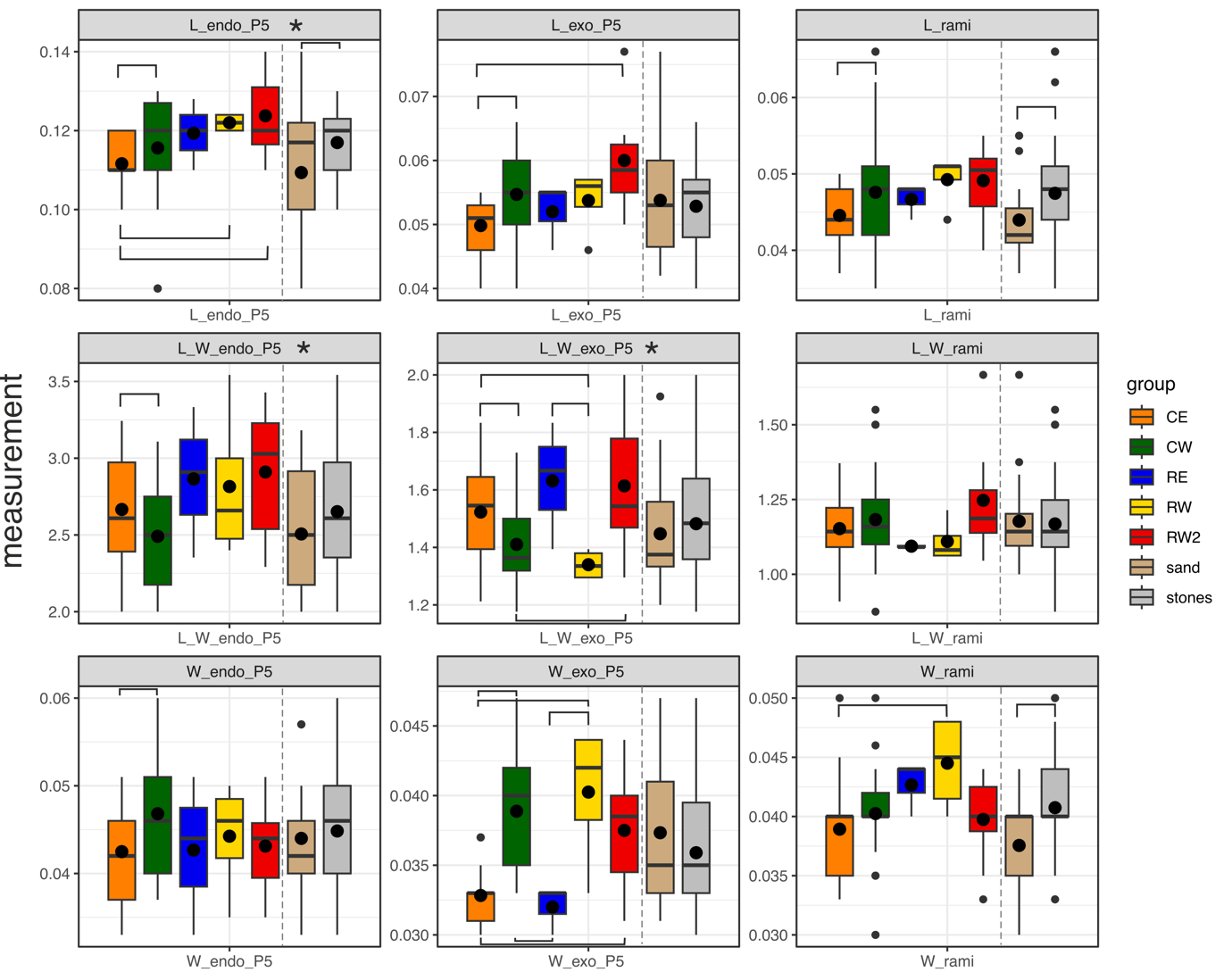


Figure S9. Panel of boxplots presenting diversity of values of each of the morphometric measurements and for the length-width ratios, for data grouped by the COI molecular lineages (CE, CW, RW, RW2, RE) and by type of substrate (sand and stones). The boxes represent the interquartile range, horizontal lines within boxes – median, dots within boxes – mean, dots outside boxes – outliers, vertical lines – range of variation. Brackets connect pairs of boxplots which show a significant difference in a pairwise Tukey test. The asterisks indicate significant interaction of the classifying factors (molecular lineage, type of substrate) in two-way ANOVA. Abbreviations are as follows: L_endo, length of P5 endopod; W_endo, width of P5 endopod; L_W_endo, ratio of length to width of P5 endopod; ; L_exo, length of P5 exopod; W_exo, width of P5 exopod; L_W_exo, ratio of length to width of P5 exopod; L_rami, length of caudal ramus; W_rami, width of caudal ramus, L_W_rami, ratio of length to width of caudal ramus (see Figure 5 for definitions).


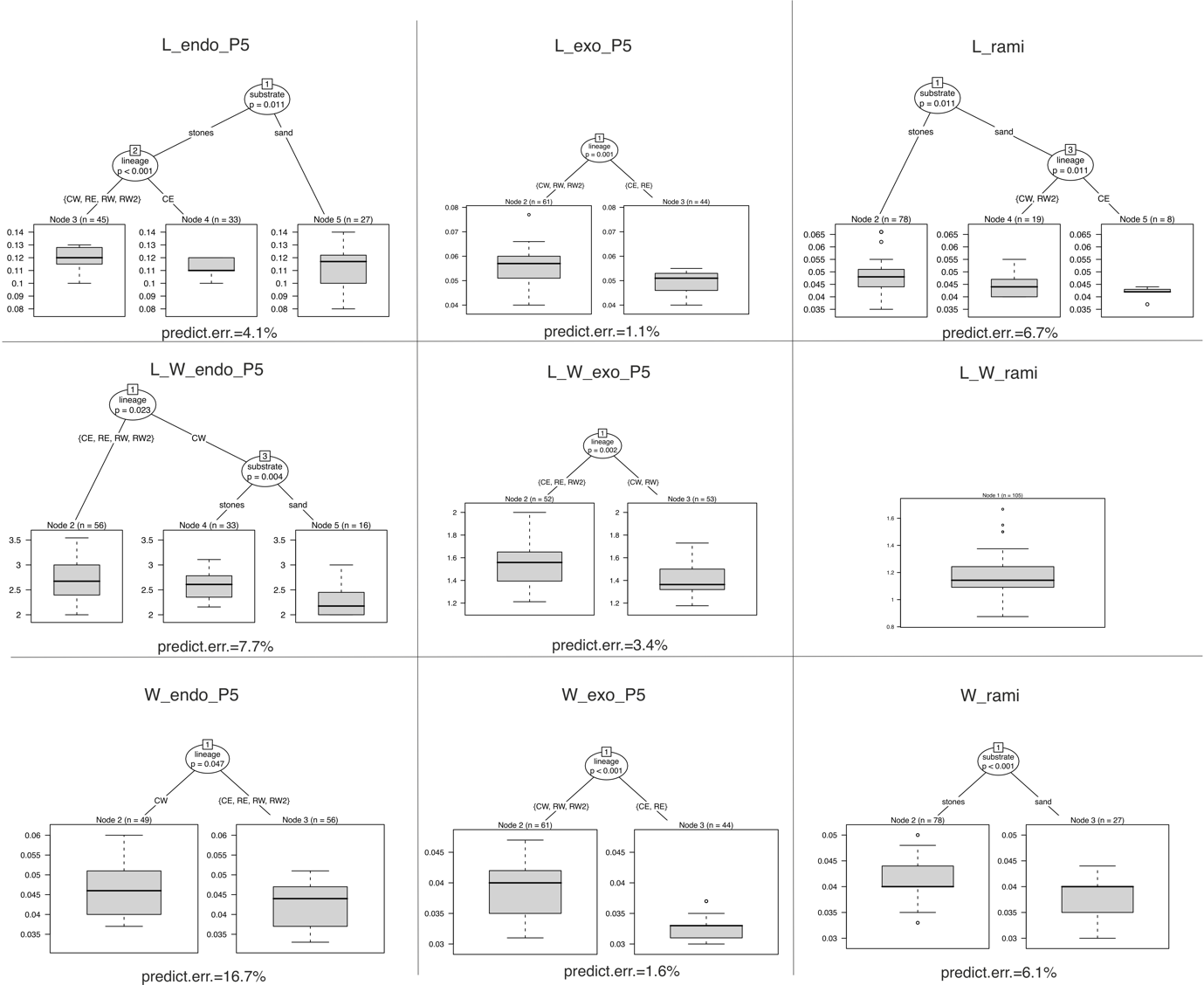


Figure S10. Conditional inference trees from random forest analysis (Liaw & Wiener, 2002) of morphometric variation, for data grouped by the molecular lineage and by substrate type. Numbers in boxes at the top are hierarchical node numbers. Boxplots show the variation of each measurement in the terminal groups, *n* is the number of specimens in those groups.

Liaw A, Wiener M (2002). “Classification and Regression by randomForest.” *R News*, 2(3), 18-22. [https://CRAN.R-project.org/doc/Rnews/](https://cran.r-project.org/doc/Rnews/).


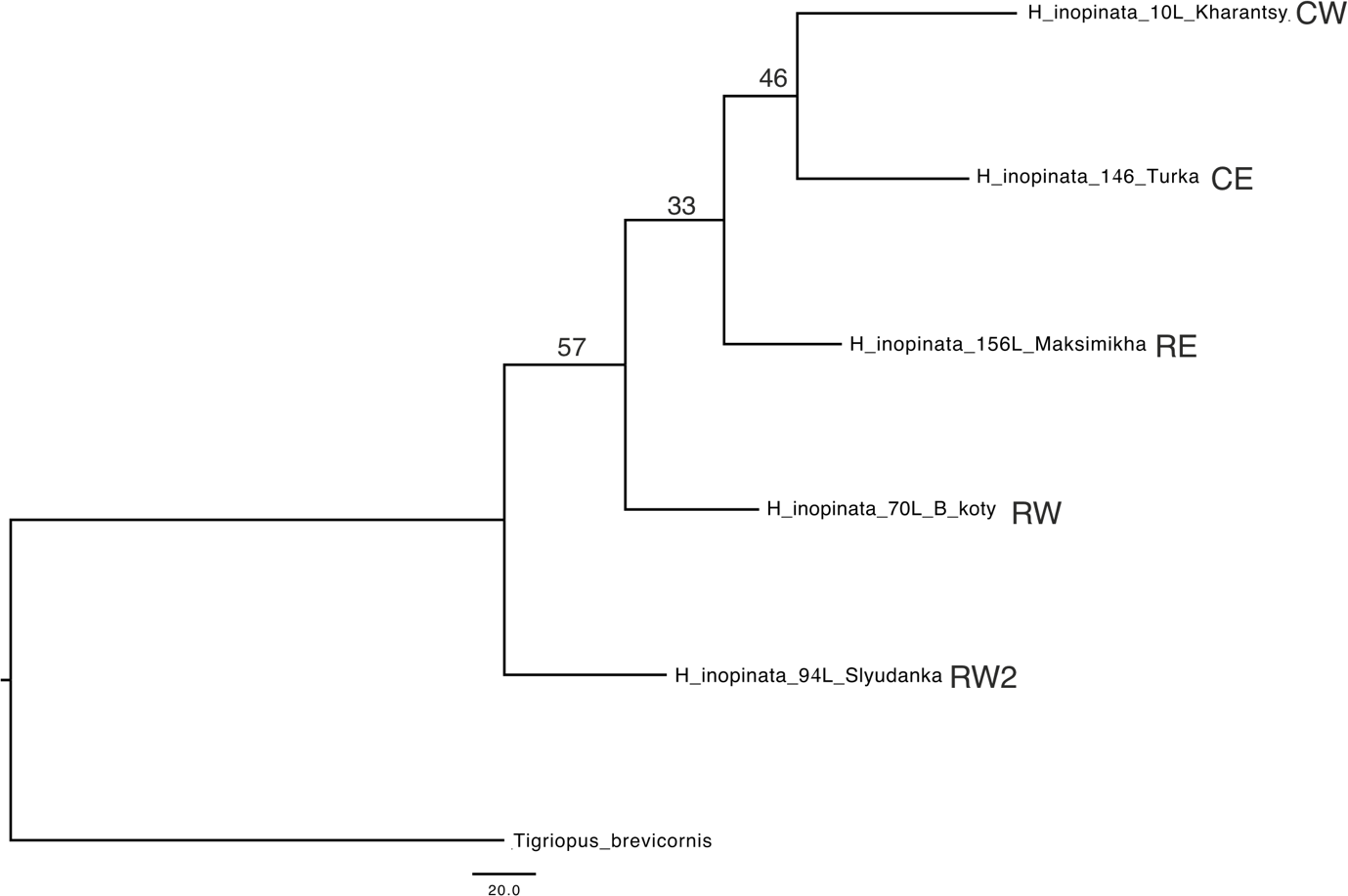


Figure S11. Maximum parsimony tree from concatenated three-locus sequences (COI+ITS1+28S) of representative individuals from each of the five *H. inopinata* lineages. The tree was built in PAUP using ACCTRAN optimization; tree scale indicates number of difference Numbers at the nodes are bootstrap values. As an outgroup sequences of *Tigriopus brevicornicus* were used (EF207720, KF851273, AY598461).


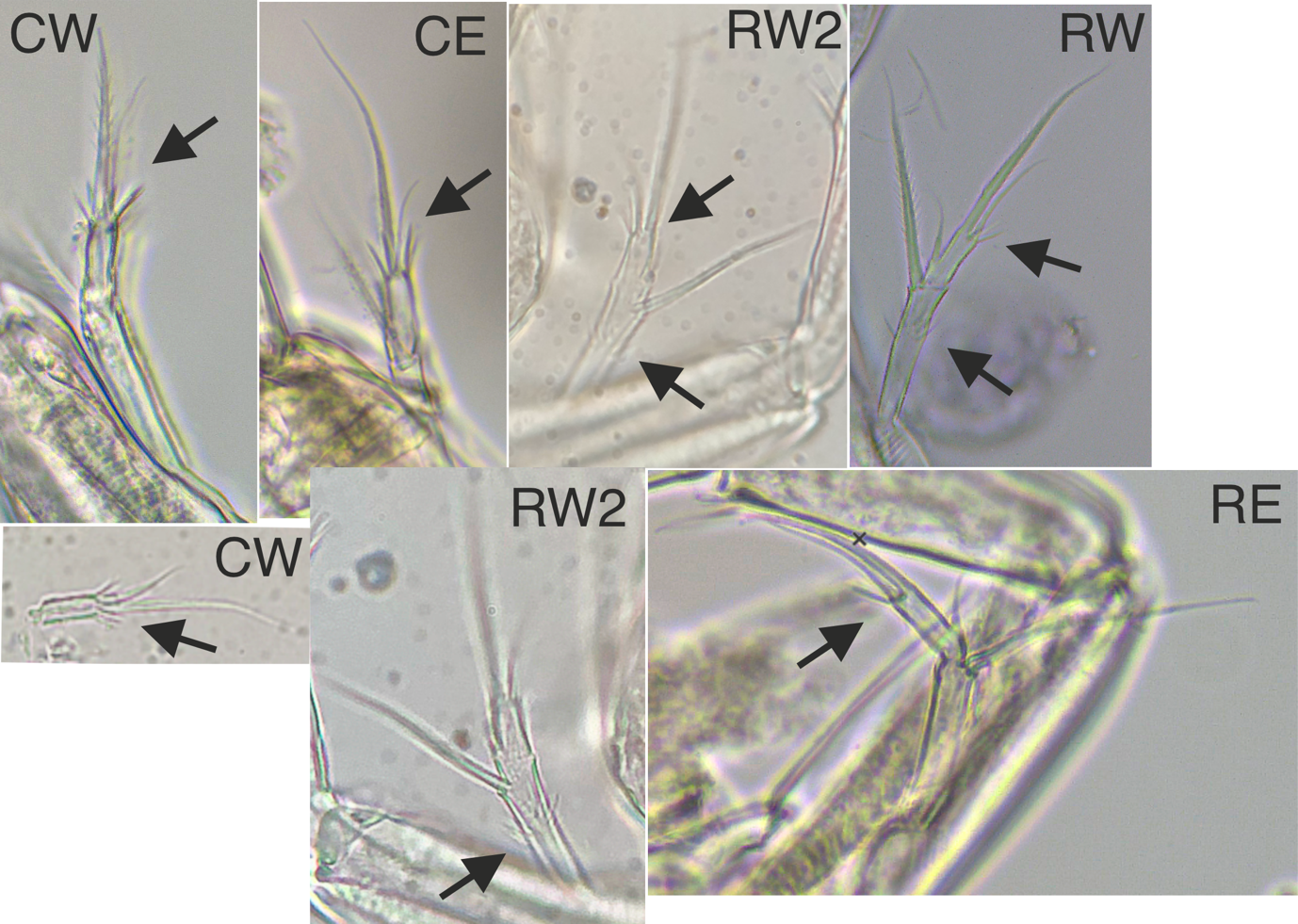


Figure S12. Photographs of Antenna II exopodite from different *H. inopinata* lineages. CW – specimen (7) from Kharantsy, Olkhon island, CE –Barguzin (162), RW2 – Sakhyurta (9a), RW – Kotelnikovskii Mys (3kt), RE –Malyi Ushkanii island (6U).


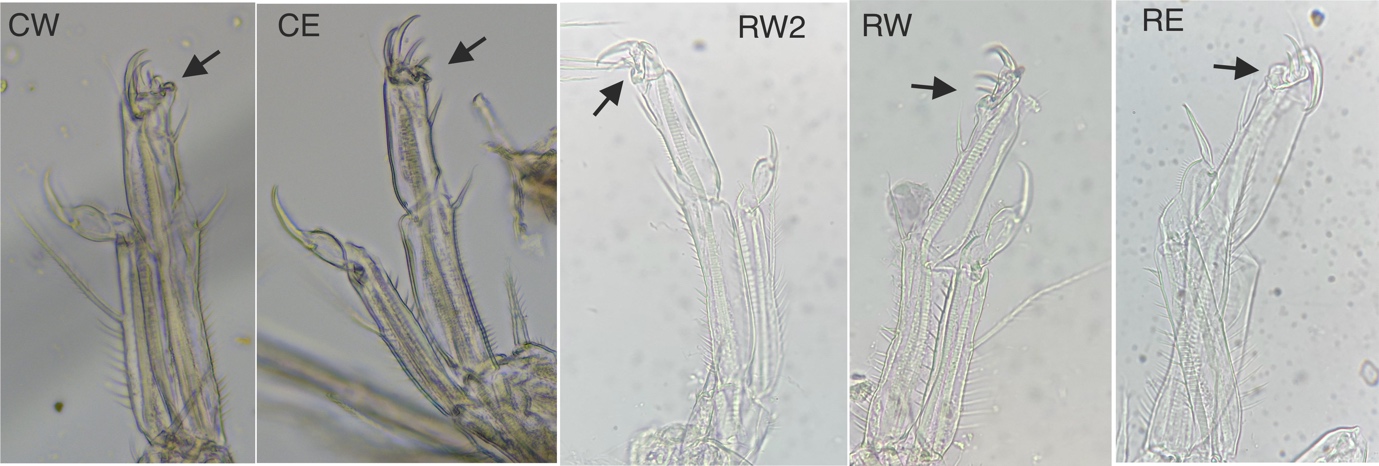


Figure S13. Photographs of Maxillipeds (P1) from different *H. inopinata* lineages. CW – specimen (7) from Kharantsy, Olkhon island, CE –Barguzin (162), RW2 – Sakhyurta (9a), RW – Kotelnikovskii Mys (3kt), RE –Malyi Ushkanii island (6U).
